# Characterization of high-grade prostate cancer at multiparametric MRI: assessment of PI-RADS version 2.1 and version 2 descriptors across 21 readers with varying experience (MULTI study)

**DOI:** 10.1186/s13244-023-01391-z

**Published:** 2023-03-20

**Authors:** Florian Di Franco, Rémi Souchon, Sébastien Crouzet, Marc Colombel, Alain Ruffion, Amna Klich, Mathilde Almeras, Laurent Milot, Muriel Rabilloud, Olivier Rouvière, on behalf of the MULTI Study Group, Sabine Debeer, Marine Dubreuil-Chambardel, Stéphanie Bravetti, Stéphane Cadot, Bénédicte Cayot, Paul-Hugo Jouve de Guibert, Paul Cezar Moldovan, Gaele Pagnoux, Clément Pernet, Louis Perrier, Nicolas Stacoffe, Sarah Transin, Michel Abihanna, Sébastien Ronze, Alexandre Ben Cheikh, Flavie Bratan, Rémy Rosset, Domitille Cadiot, Leangsing Iv, Jean Champagnac, Nicolas Girouin, Olivier Lopez, Athivada Soto Thammavong

**Affiliations:** 1 Hospices Civils de Lyon, Department of Imaging, Hôpital Edouard Herriot, 69437 Lyon, France; 2 INSERM, LabTau, U1032, Lyon, France; 3 Université de Lyon, Université Lyon 1, Lyon, France; 4 Faculté de Médecine Lyon Est, Lyon, France; 5 Hospices Civils de Lyon, Department of Urology, Hôpital Edouard Herriot, 69437 Lyon, France; 6 Hospices Civils de Lyon, Department of Urology, Centre Hospitalier Lyon Sud, Pierre-Bénite, France; 7 Equipe 2—Centre d’Innovation en Cancérologie de Lyon, 3738 Lyon, EA France; 8 Faculté de Médecine Lyon Sud, 69003 Lyon, France; 9 Service de Biostatistique et Bioinformatique, Hospices Civils de Lyon, Pôle Santé Publique, 69003 Lyon, France; 10 UMR 5558, Laboratoire de Biométrie et Biologie Évolutive, CNRS, Équipe Biostatistique-Santé, 69100 Villeurbanne, France

**Keywords:** Prostatic neoplasms, Magnetic resonance imaging, Observer variation, Image-guided biopsy

## Abstract

**Objective:**

To assess PI-RADSv2.1 and PI-RADSv2 descriptors across readers with varying experience.

**Methods:**

Twenty-one radiologists (7 experienced (≥ 5 years) seniors, 7 less experienced seniors and 7 juniors) assessed 240 ‘predefined’ lesions from 159 pre-biopsy multiparametric prostate MRIs. They specified their location (peripheral, transition or central zone) and size, and scored them using PI-RADSv2.1 and PI-RADSv2 descriptors. They also described and scored ‘additional’ lesions if needed. Per-lesion analysis assessed the ‘predefined’ lesions, using targeted biopsy as reference; per-lobe analysis included ‘predefined’ and ‘additional’ lesions, using combined systematic and targeted biopsy as reference. Areas under the curve (AUCs) quantified the performance in diagnosing clinically significant cancer (csPCa; ISUP ≥ 2 cancer). Kappa coefficients (*κ*) or concordance correlation coefficients (CCC) assessed inter-reader agreement.

**Results:**

At per-lesion analysis, inter-reader agreement on location and size was moderate-to-good (*κ* = 0.60–0.73) and excellent (CCC ≥ 0.80), respectively. Agreement on PI-RADSv2.1 scoring was moderate (*κ* = 0.43–0.47) for seniors and fair (*κ* = 0.39) for juniors. Using PI-RADSv2.1, juniors obtained a significantly lower AUC (0.74; 95% confidence interval [95%CI]: 0.70–0.79) than experienced seniors (0.80; 95%CI 0.76–0.84; *p* = 0.008) but not than less experienced seniors (0.74; 95%CI 0.70–0.78; *p* = 0.75). As compared to PI-RADSv2, PI-RADSv2.1 downgraded 17 lesions/reader (interquartile range [IQR]: 6–29), of which 2 (IQR: 1–3) were csPCa; it upgraded 4 lesions/reader (IQR: 2–7), of which 1 (IQR: 0–2) was csPCa. Per-lobe analysis, which included 60 (IQR: 25–73) ‘additional’ lesions/reader, yielded similar results.

**Conclusions:**

Experience significantly impacted lesion characterization using PI-RADSv2.1 descriptors. As compared to PI-RADSv2, PI-RADSv2.1 tended to downgrade non-csPCa lesions, but this effect was small and variable across readers.

**Supplementary Information:**

The online version contains supplementary material available at 10.1186/s13244-023-01391-z.

## Key points

1. Juniors characterized aggressive cancers less well than experienced seniors on prostate MRI.
2. Agreement between readers remained moderate even for experienced readers.
3. As compared to version 2, PI-RADSv2.1 descriptors tended to show improved specificity.

**Supplementary Information:**

The online version contains supplementary material available at 10.1186/s13244-023-01391-z.

## Introduction

Interpretation of prostate multiparametric magnetic resonance imaging (MRI) is challenging because of potential discordance between findings from the different pulse sequences and substantial overlap between the appearance of benign and malignant conditions. These difficulties led to the creation of the Prostate Imaging-Reporting and Data System (PI-RADS). For each pulse sequence, semi-objective descriptors are used to classify lesions into specific categories. These categories are then combined into a final score assessing the likelihood of clinically significant prostate cancer (csPCa). PI-RADS version 2 (PI-RADSv2) showed good performance but moderate inter-reader agreement [1–9]. Version 2.1 (PI-RADSv2.1) was published in 2019 to address PI-RADSv2 limitations and improve reproducibility by clarifying some descriptors [10].

Although PI-RADSv2.1 has been extensively evaluated [11–22], meta-analyses yielded discordant results on the relative diagnostic performance of PI-RADSv2 and PI-RADSv2.1 [23–25]. Particularly, whether PI-RADSv2.1 improves inter-reader agreement remains unclear.

MRI interpretation can be broken down into two phases: the detection phase, in which the radiologist sees the lesion, and the characterization phase, in which they assess its degree of suspicion. Each phase contributes to the scoring performance and variability.

In this study, we focussed on the characterization phase by asking 21 readers with varying experience to assess, using PI-RADSv2.1 and PI-RADSv2 descriptors, the same set of MRI lesions with known histology. Our primary objective was to determine whether these descriptors were precise enough to allow readers to assign similar scores to the same lesions.

## Materials and methods

### Prospective biopsy database

As of September 2008, consecutive patients undergoing prostate MRI and subsequent biopsy at our institution were included in a prospective database after signing institutional review board-approved consent forms [26]. MRIs combined T2-weighted (T2w), diffusion-weighted (Dw) and dynamic contrast-enhanced (DCE) imaging at 1.5 T or 3 T. Transrectal biopsies combined systematic and targeted cores obtained under cognitive or MRI/ultrasound fusion (Urostation, Koelis) depending on the lesions’ location and the operator’s preference. Two to five targeted cores were taken from each lesion and at least two systematic cores (one paramedian, one lateral) from each PZ sextant. The operator could omit systematic cores from PZ sextants with lesions targeted at biopsy. TZ was biopsied only if it contained suspicious lesions.

### Readers

Twenty-one radiologists (14 seniors, 7 juniors), from nine different private and public hospitals, participated in the study. Seven seniors (experienced seniors) had more than 5 years and seven (less experienced seniors) less than 5 years of experience. Four juniors had achieved a 6-month rotation in a department of uroradiology, three had passed an advanced diploma in genitourinary imaging, and two had no experience in prostate imaging (Additional file 1: I). Before starting the study, juniors took a 2-h class on PI-RADS scoring. Then, all readers attended a meeting during which representative cases were presented and differences between PI-RADSv2 and PI-RADSv2.1 were discussed.

### Study sample

Consecutive biopsy-naïve patients included in the biopsy database between September 2015 and July 2016 were retrospectively selected. September 2015 corresponded to the date of implementation of PI-RADSv2 guidelines at our institution (Additional file 1: II). July 2016 was chosen to allow for at least four years of follow-up. These dates were also chosen because during that period, biopsy operators were instructed to target all focal lesions, even those with a low degree of suspicion, resulting in a large variety of targeted lesions.

Readers were given a four-month period (September-December 2019) to interpret the MRIs of the study sample. They were blinded to clinical and histological data, and to each other’s assessment.

### Predefined lesions

First, readers assessed the ‘predefined lesions’, i.e. the MRI lesions targeted at biopsy. These were indicated on one T2w image. Readers were informed that, at the time the sample was acquired, biopsy operators were instructed to target all focal lesions, and thus, that a substantial proportion of the predefined lesions was expected to be benign. Nonetheless, the proportion of benign lesions and csPCas in the sample was not disclosed.

Readers noted the lesions’ maximal diameter, side and location (PZ, TZ or central zone (CZ)). When lesions extended into several zones, the zone in which most of the lesion was located was selected.

Then, readers defined the lesions’ PI-RADSv2 and PI-RADSv2.1 categories, for each pulse sequence, following as closely as possible the manual definitions of these categories (Additional file 1: II). The lesions’ final PI-RADSv2 and PI-RADSv2.1 scores were automatically calculated based on their location, size and pulse sequence categories.

### Additional lesions

If needed, readers could note additional lesions that had not been targeted at biopsy. They defined, for each ‘additional lesion’, its location, diameter and pulse sequence categories according to PI-RADSv2 and PI-RADSv2.1 manual definitions. The overall scores were automatically calculated.

### Per-lobe and per-patient scores

The PI-RADSv2 and PI-RADSv2.1 scores of each prostate lobe/patient were computed by selecting the highest scores of the predefined and additional lesions described in this lobe/patient. Lobes or patients with no lesion received default PI-RADSv2 and PI-RADSv2.1 scores of 1 (Additional file 1: III).

### Follow-up

Follow-up data were retrieved in June–September 2020. The medical files of the patients without csPCa at initial biopsy were searched for any additional prostate biopsy performed during follow-up. Patients without follow-up at our institution were contacted by telephone or through their general practitioner.

### Reference standard and csPCa definition

For characterizing predefined lesions, targeted biopsy findings were used as reference standard. For per-lobe and per-patient analysis that took into account predefined and additional lesions, combined targeted and systematic biopsy findings were used as reference standard. csPCa was defined as International Society of Urological Pathology (ISUP) grade ≥ 2 cancer.

### Statistical analysis

Quantitative characteristics were described using medians and interquartile ranges (IQRs). Qualitative characteristics were described using absolute and relative frequencies.

A mixed probit regression corresponding to the binormal model was used to model the receiver operating characteristic (ROC) curves according to the reader’s experience, with the reader as random effect [27, 28]. Regression coefficients for experienced and less experienced seniors in comparison to juniors allowed to quantify and test the effect of reader’s experience on the diagnostic performance of the scores. The model was also used to predict the ROC curve for each category of readers. Areas under the curve (AUCs) were estimated using the binormal method [28]. Stratified bootstrap with sampling at the level of patients within strata defined by the presence or absence of csPCA was used to build AUCs 95% confidence intervals (CIs). A logistic mixed model was used to model sensitivity and specificity according to the reader’s experience, with the reader as random effect. Sensitivities and specificities were estimated with their 95% CIs for predefined thresholds of PI-RADS scores of ≥ 3 and ≥ 4. Inter-reader agreement was estimated using Cohen’s kappa coefficient (*κ*) for location and DCE categories, concordance correlation coefficient for size, and weighted *κ* for T2w and Dw categories and overall scores. Coefficients of ≤ 0.20, 0.21–0.40, 0.41–0.60, 0.61–0.80 and > 0.80 indicate poor, fair, moderate, good and excellent agreement, respectively.

Similar analyses were performed at lobe and patient level. R software, version 3.6.1 (https://cran.r-project.org) was used for analysis. This study is registered with ClinicalTrials.gov, number NCT04299997.

## Results

### Study sample

**Fig. 1:**
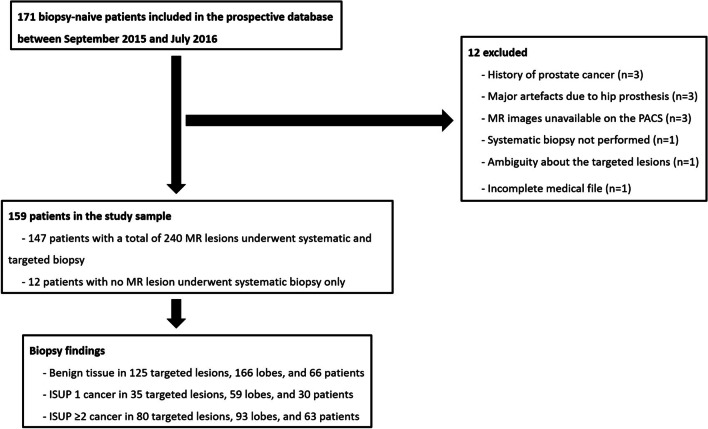
Standards for reporting of diagnostic accuracy (STARD) flow diagram. *MR* Magnetic resonance, *PACS* Picture archiving communication system, *ISUP* International society of urological pathology

A total of 159 patients imaged at 1.5 T (*n =* 77) or 3 T (*n =* 82) were included (Fig. 1, Table 1). MRI scanners and protocols are detailed in Additional file 1: IV. Twelve patients had normal MRI, and 240 lesions were targeted in the 147 remaining patients. These 240 lesions constituted the ‘predefined lesions’ corpus.

**Table 1:** Patients’ characteristics

Clinical data	Median age (years)			67 (61–71)
	Median PSA (ng/mL)			8 (6–11)
	Median PSA density (ng/mL/mL)			0.17 (0.12–0.26)
	Digital rectal examination	T1c		81 (50.3%)
		T2		39 (25.8%)
		T3		3 (2%)
		Missing data		36 (21.8%)
Prostate biopsy	Median delay between MRI and biopsy (days)			35 (0–59)
	Median number of biopsy samples per patient			15 (14–17)
	Biopsy results ^(1)^	Predefined lesions (*n =* 240)	Benign findings	125 (52%)
			ISUP1 cancer	35 (15%)
			ISUP ≥ 2 cancer	80 (33.3%)
		Per-lobe analysis (*n =* 318)	Benign findings	166 (52%)
			ISUP1 cancer	59 (19%)
			ISUP ≥ 2 cancer	93 (29.2%)
		Per-patient analysis (*n =* 159)	Benign findings	66 (41%)
			ISUP1 cancer	30 (19%)
			ISUP ≥ 2 cancer	63 (39.6%)

Data are median (interquartile range) or *n* (%)

(1) Results of targeted biopsy for predefined lesions and of combined systematic and targeted biopsy for per-lobe and per-patient analyses

*PSA* Prostate-specific antigen, *MRI* Magnetic resonance imaging, *ISUP* International society of urological pathology

### Predefined lesions

#### Agreement on location, size and PI-RADS categories

Agreement on lesions’ location was moderate-to-good (*κ* = 0.60–0.73), with experienced seniors obtaining the highest *κ* (Table 2). Perfect agreement across all readers was reached in only 142/240 lesions (PZ, *n =* 133; TZ, *n =* 9; CZ, *n =* 0). Depending on the reader, a median number of 204 (IQR, 202–210), 26 (IQR, 23–28) and 10 (IQR, 6–12) lesions were localized in PZ, TZ and CZ, respectively (Additional file 1: V.1). Agreement on size was excellent (CCC ≥ 0.80) for all groups of readers (Table 2).

**Table 2:** Inter-reader agreement (analysis of the 240 predefined lesions)

		All readers	Experienced seniors	Less experienced seniors	Juniors
Lesion location (PZ, TZ or CZ) ^(1)^		0.65 [0.59–0.70]	0.73 [0.67–0.79]	0.60 [0.54–0.65]	0.63 [0.55–0.69]
Lesion size ^(2)^		0.80 [0.75–0.86]	0.82 [0.76–0.88]	0.82 [0.76–0.88]	0.81 [0.76–0.87]
PI-RADSv2.1 categories	T2w ^(3)^	0.49 [0.45–0.52]	0.58 [0.54–0.62]	0.51 [0.46–0.55]	0.42 [0.38–0.46]
	Dw ^(3)^	0.51 [0.47–0.54]	0.57 [0.53–0.61]	0.52 [0.47–0.56]	0.48 [0.44–0.52]
	DCE ^(1)^	0.30 [0.25–0.34]	0.30 [0.25–0.35]	0.29 [0.21–0.35]	0.38 [0.33–0.42]
PI-RADSv2 categories	T2w ^(3)^	0.50 [0.47–0.53]	0.59 [0.55–0.64]	0.53 [0.49–0.58]	0.43 [0.39–0.47]
	Dw ^(3)^	0.53 [0.49–0.56]	0.56 [0.53–0.60]	0.54 [0.50–0.58]	0.52 [0.48–0.56]
	DCE ^(1)^	0.31 [0.26–0.35]	0.31 [0.26–0.36]	0.31 [0.22–0.37]	0.38 [0.33–0.43]
PI-RADSv2.1 score ^(3)^		0.42 [0.38–0.45]	0.47 [0.43–0.51]	0.43 [0.39–0.48]	0.39 [0.35–0.43]
PI-RADSv2 score ^(3)^		0.44 [0.40–0.47]	0.45 [0.41–0.49]	0.45 [0.41–0.49]	0.44 [0.40–0.49]

(1) Data are presented as mean kappa coefficient [95% confidence interval]

(2) Data are presented as mean concordance correlation coefficients [95% confidence interval]

(3) Data are presented as mean weighted kappa coefficients [95% confidence interval]

*PZ* Peripheral zone, *TZ* Transition zone, *CZ* Central zone, *T2w* T2-weighted, *Dw* Diffusion-weighted, *PI-RAD*S Prostate imaging-reporting and data system

Agreement on PI-RADSv2.1 T2w and Dw categories was moderate (*κ* = 0.42–0.58 and *κ* = 0.48–0.57, respectively) and tended to increase with experience. For DCE categories, agreement was fair (*κ* = 0.30–0.38) for all groups of readers. Similar findings were obtained with PI-RADSv2 categories (Table 2).

#### PI-RADS scores

Inter-reader agreement for PI-RADSv2.1 scoring was moderate for seniors (*κ* = 0.43–0.47) and fair for juniors (*κ* = 0.39; Table 2). Using PI-RADSv2.1, juniors obtained a significantly lower AUC (0.74 [95%CI, 0.70–0.79]) than experienced seniors (0.80 [95%CI, 0.76–0.84], *p* = 0.008), but not than less experienced seniors (0.74 [95%CI, 0.70–0.78], *p* = 0.75). Experienced seniors tended to show higher specificity, but the difference was not statistically significant (Tables 3–4, Additional file 1: V.2-V.5).

**Table 3:** PI-RADSv2.1 and PI-RADSv2 scores assigned by the three groups of readers

			Benign lesions & ISUP 1 cancers			csPCas		
			Experienced seniors	Less experienced seniors	Juniors	Experienced seniors	Less experienced seniors	Juniors
Predefined lesions (*N =* 240)	PI-RADSv2.1	1	4 [2–12]	4 [1–5]	8 [2–9]	1 [0–2]	1 [0–1]	1 [0–1]
		2	49 [20–61]	20 [14–39]	22 [19–52]	5 [3–6]	0 [0–2]	4 [2–9]
		3	24 [18–29]	15 [12–18]	33 [28–34]	5 [4–7]	4 [3–6]	8 [3–11]
		4	65 [44–86]	96 [90–111]	73 [56–95]	35 [34–39]	39 [38–41]	35 [34–39]
		5	11 [8–15]	13 [11–20]	15 [10–24]	32 [28–36]	33 [30–35]	32 [26–35]
	PI-RADSv2.0	1	3 [1–10]	2 [1–3]	2 [1–7]	1 [0–3]	0 [0–0]	0 [0–1]
		2	28 [17–41]	23 [12–24]	21 [12–23]	3 [2–5]	1 [0–4]	2 [1–3]
		3	26 [21–32]	16 [12–18]	38 [29–44]	5 [5–7]	3 [2–4]	10 [3–12]
		4	87 [49–97]	105 [100–112]	78 [71–97]	38 [34–40]	40 [38–43]	41 [37–42]
		5	11 [9–15]	13 [11–19]	15 [11–24]	32 [28–36]	34 [31–35]	32 [26–35]
Per-lobe analysis (*N =* 318)	PI-RADSv2.1	1	71 [7–78]	49 [8–58]	50 [22–57]	4 [0–7]	1 [0–3]	4 [2–6]
		2	57 [48–60]	49 [21–75]	40 [37–61]	5 [4, 5]	3 [2–7]	4 [2–5]
		3	33 [22–33]	17 [14–18]	37 [32–47]	5 [3–6]	5 [2–10]	8 [3–10]
		4	60 [46–88]	99 [89–108]	77 [58–96]	36 [32–39]	37 [37–39]	36 [35–38]
		5	18 [11–23]	15 [12–23]	19 [15–35]	43 [36–49]	42 [39–44]	43 [35–49]
	PI-RADSv2.0	1	1 [0–2]	1 [0–2]	1 [0–3]	0 [0–1]	0 [0–0]	0 [0–1]
		2	107 [77–114]	82 [63–89]	65 [58–83]	6 [5–11]	7 [5–9]	6 [4–8]
		3	31 [22–39]	15 [11–23]	37 [28–51]	5 [3–6]	3 [2–4]	8 [3–10]
		4	79 [54–101]	106 [102–112]	81 [72–98]	39 [31–41]	38 [36–41]	38 [36–40]
		5	18 [11–24]	15 [12–22]	19 [15–35]	43 [36–49]	42 [42–46]	43 [36–49]

Data are presented as median number of lesions/lobes [interquartile range]

*N* Number of lesions/lobes, *csPCas* Clinically significant prostate cancers, *ISUP *International society of urological pathology, *PI-RADS* prostate imaging-reporting and data system

**Table 4:** Sensitivities and specificities obtained by the three groups of readers using PI-RADSv2.1 and PI-RADSv2 scoring

			Threshold ≥ 3				Threshold ≥ 4			
			Se [95% CI]	*p*^(1)^	Sp [95% CI]	*p*^(2)^	Se [95% CI]	*p*^(1)^	Sp [95% CI]	p^(2)^
Predefined lesions (*N =* 240)	PI-RADSv2.1	Experienced seniors	93 [85–97]	0.61	33 [19–52]	0.15	85 [76–92]	0.97	51 [36–65]	0.36
		Less experienced seniors	98 [95–99]	0.12	13 [7–25]	0.44	91 [85–95]	0.19	26 [16–40]	0.10
		Juniors	94 [88–98]	-	19 [10–33]	-	86 [76–92]		42 [28–56]	-
	PI-RADSv2	Experienced seniors	94 [89–97]	0.08	24 [15–38]		87 [79–92]	0.74	45 [31–59]	0.06
		Less experienced seniors	98 [95–99]	0.66	11 [6–20]		93 [88–96]	0.18	24 [15–36]	0.87
		Juniors	97 [95–99]	-	12 [7–21]		88 [81–93]	-	36 [24–50]	-
Per-lobe analysis (*N =* 318)	PI-RADSv2.1	Experienced seniors	90 [86–94]	0.63	48 [39–58]	0.08	85 [78–90]	0.90	62 [51–72]	0.34
		Less experienced seniors	92 [88–95]	0.84	38 [29–47]	0.88	87 [81–92]	0.44	48 [37–59]	0.39
		Juniors	92 [87–95]	-	37 [28–46]	-	84 [78–90]	-	55 [44–66]	-
	PI-RADSv2	Experienced seniors	91 [88–94]	0.34	42 [34–50]	0.06	87 [81–91]	0.99	56 [46–67]	0.36
		Less experienced seniors	93 [90–95]	0.73	34 [27–43]	0.54	89 [84–93]	0.54	45 [35–56]	0.55
		Juniors	93 [90–95]	-	31 [24–39]	-	87 [81–91]	-	50 [39–60]	-

(1) *p* value comparing the sensitivity to that of junior readers

(2) *p* value comparing the specificity to that of junior readers

Sensitivities and specificities are expressed in percentages

Similar findings were obtained with PI-RADSv2 (Tables 2–4, Additional file 1: V.2–V.5). All groups of readers tended to assign lower scores to non-csPCa lesions using PI-RADSv2.1 than using PI-RADSv2. As compared to PI-RADSv2, PI-RADSv2.1 downgraded a median number of 17 lesions per reader (IQR, 6–29), of which 2 (IQR, 1–3) were csPCa. It upgraded a median number of 4 lesions per reader (IQR, 2–7), of which 1 (IQR, 0–2) was csPCa. The most frequent downgradings were from PI-RADS scores of 3 to 2 and 4 to 2. In TZ, a median number of 2 lesions (IQR, 0–2) were downgraded from a score of 3 to 2, and a median number of 1 lesion (IQR, 0–2) was upgraded from a score of 2 to 3 (Additional file 1: V.6-V.7).

### Additional lesions

Readers described a median number of 60 ‘additional lesions’ (IQR, 25–73; Additional file 1: VI.1).

### Per-lobe and per-patient scores

At per-lobe analysis, after taking into consideration predefined and additional lesions, inter-reader agreement for PI-RADSv2.1 scoring was moderate-to-good (*κ* = 0.54–0.63; Table 5). Using PI-RADSv2.1, juniors obtained a significantly lower AUC (0.79 [95%CI, 0.75–0.83]) than experienced seniors (0.82 [95%CI, 0.79–0.86], *p* = 0.03), but not than less experienced seniors (0.79 [95%CI, 0.76–0.83], *p* = 0.71). Experienced seniors tended to show higher specificity, but the difference was not statistically significant (Table 5, Additional file 1: VI.2–VI.5).

**Table 5:** Inter-reader agreement (per-lobe analysis)

PI-RADSv2.1	
All readers	0.56 [0.53–0.59]
Experienced seniors	0.55 [0.51–0.59]
Less experienced seniors	0.63 [0.59–0.66]
Juniors	0.54 [0.51–0.58]
PI-RADSv2	
All readers	0.58 [0.55–0.61]
Experienced seniors	0.56 [0.52–0.60]
Less experienced seniors	0.65 [0.62–0.68]
Juniors	0.57 [0.54–0.61]

Data are presented as mean weighted kappa coefficients [95% confidence interval]

**Fig. 2:**
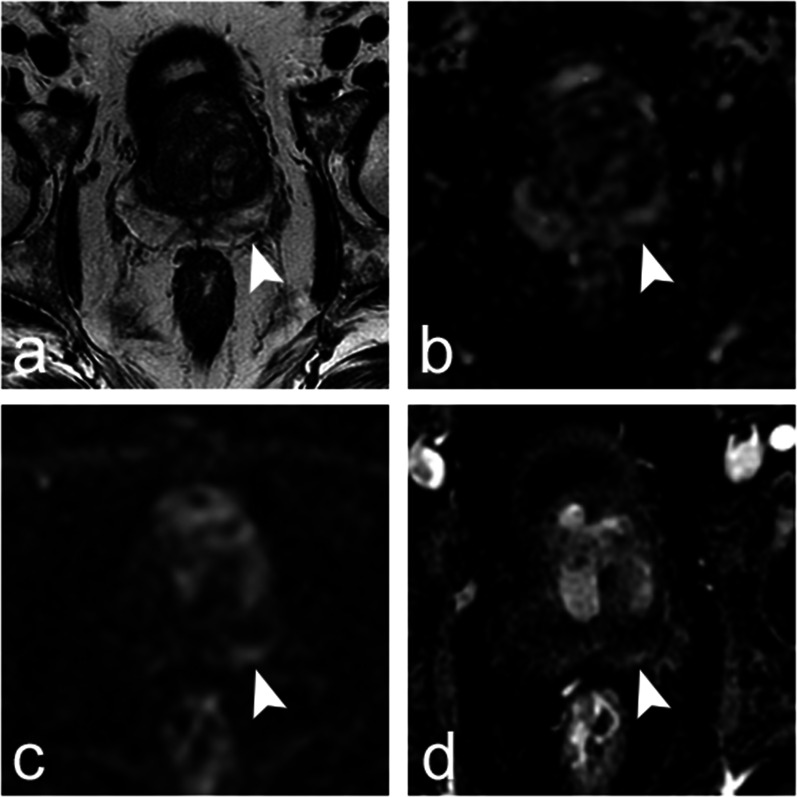
Axial images obtained in a 62-year-old patient with prostate-specific antigen (PSA) level of 8.1 ng/mL and normal digital rectal examination. Prostate multiparametric magnetic resonance imaging (**a**, T2-weighted image; **b**, apparent diffusion coefficient map; **c**, diffusion-weighted trace image obtained with *b* value of 2000 s/mm^2^; and **d**, dynamic contrast-enhanced image) showed a 13-mm linear lesion parallel to the capsule in the peripheral zone of the left base (**a**–**d**, arrowheads). Using PI-RADSv2 descriptors, 17 readers assigned to the lesion a T2-weighted imaging (T2WI) category of 2 (‘Linear, wedge-shaped or diffuse mild hypointensity, usually indistinct margin’), two readers a T2WI category of 3 (‘Heterogeneous signal intensity or non-circumscribed, rounded, moderate hypointensity’) and two readers a T2WI category of 4 (‘Circumscribed, homogeneous moderate hypointense focus/mass confined to prostate and < 1.5 cm in greatest dimension’). Two readers assigned a diffusion-weighted imaging (DWI) category of 2 (‘Indistinct hypointense on ADC ‘), fifteen readers a DWI category of 3 (‘Focal mildly/moderately hypointense on ADC and isointense/mildly hyperintense on high b value DWI’) and three readers a DWI category of 4 (‘Focal markedly hypointense on ADC and markedly hyperintense on high b value DWI < 1.5 cm on axial images’). Seventeen readers judged the lesion as positive at dynamic contrast-enhanced (DCE) imaging (‘Focal, AND earlier than or contemporaneously with enhancement of adjacent tissues, AND corresponds to suspicious findings on T2WI and/or DWI’), and four readers judged it as negative (‘No early enhancement, OR diffuse enhancement not corresponding to a focal finding on T2W and/or DWI, OR focal enhancement corresponding to a lesion demonstrating features of BPH on T2W’). The final PI-RADSv2 score was 2 for three readers, 3 for four readers and 4 for fourteen readers. Using PI-RADSv2.1 descriptors, the assignment of T2WI categories was the same as with PI-RADSv2 since the descriptors are identical. Fifteen readers assigned a DWI category of 2 (‘Linear/wedge-shaped hypointense on ADC and/or linear/wedge-shaped hyperintense on high b value DWI’), four readers a diffusion category of 3 (‘Focal (discrete and different from the background) hypointense on ADC and/or focal hyperintense on high b value DWI; may be markedly hypointense on ADC or markedly hyperintense on high b value DWI but not both’) and two readers a DWI category of 4 (‘Focal markedly hypointense on ADC and markedly hyperintense on high b value DWI < 1.5 cm on axial images’). Sixteen readers judged the lesion as positive at DCE imaging (‘Focal, AND earlier than or contemporaneously with enhancement of adjacent tissues, AND corresponds to suspicious findings on T2W and/or DWI’) and five as negative (‘No early or contemporaneous enhancement, OR diffuse multifocal enhancement NOT corresponding to a focal finding on T2W and/or DWI, OR focal enhancement corresponding to a lesion demonstrating features of BPH on T2W, including features of extruded BPH in the PZ). The final PI-RADSv2.1 score was 2 for sixteen readers, 3 for one reader and 4 for four readers. Systematic and targeted biopsy showed normal prostate tissue, with mild inflammation in the left base. Fifty-six months later, the patient had a PSA level of 6 ng/ml and had not undergone another prostate biopsy.

Similar findings were obtained with PIRADSv2 (Tables 3–5, Additional file 1: VI.2–VI.5). As compared to PI-RADSv2, PI-RADSv2.1 downgraded a median number of 66 lobes per reader (IQR, 35–94), of which 6 (IQR, 1–11) contained csPCa at biopsy (Fig. 2). It upgraded a median number of 5 lobes per reader (IQR, 2–8), of which 1 (IQR, 0–2) contained csPCa. The most frequent downgradings were from PI-RADS scores of 2 to 1, 4 to 2 and 3 to 2 (Additional file 1: VI.6).

Per-patient analysis showed concordant results (Additional file 1: VII).

### Follow-up

Of the 96 patients without csPCa at initial biopsy, 7 with an ISUP 1 cancer received immediate radical treatment. During a median follow-up of 51 months (IQR, 45–55), 7 of the 88 remaining patients were diagnosed with an ISUP 2 cancer and none with an ISUP ≥ 3 cancer.

## Discussion

To specifically evaluate the characterizing value of the PI-RADSv2/v2.1 descriptors, we asked the readers to score the exact same corpus of lesions. To be clinically meaningful, this corpus had to include lesions with a large range of degrees of suspicion. Therefore, we selected consecutive patients who underwent MRI and biopsy at our institution in 2015–2016. At that time, our biopsy policy required to target all focal lesions, even those with a low degree of suspicion. Biopsy operators could omit systematic biopsy in PZ sextants that had targeted biopsy, which allowed targeting several lesions without unreasonably increasing the number of cores taken. Hence, 92.5% (147/159) of the study patients underwent targeted biopsy while the csPCa prevalence was only 39.6% and 33% at patient and lesion level retrospectively. Furthermore, in accordance with the recommendations of the time [29], MRI was not used to select patients for biopsy but only to indicate the lesions to target, which limited selection bias.

This set of predefined lesions was first used to assess inter-reader agreement on lesion size and location. Agreement on size was excellent (CCC ≥ 0.80). The overall agreement on lesion location (PZ, TZ or CZ) was moderate-to-good (*κ* = 0.60–0.73). Only 59% (142/240) of the predefined lesions were localized in the same zone by all readers. This is problematic since PZ and TZ lesions are scored differently, using different dominant sequences. Additionally, CZ lesions are also assessed differently, at least using PIRADSv2.1 descriptors [10]. Thus, any variability in lesion location can have major consequences on the final scoring agreement. Variability on lesion location can be explained by two main factors. First, due to the lack of well-defined anatomical landmarks between CZ and PZ, the number of lesions localized in CZ was highly variable from one reader to another. Second, partial volume effects in some locations (e.g. anterior horn of the PZ, extreme apex) made it difficult to distinguish between PZ lesions and TZ nodules protruding into the PZ. 3D T2w acquisitions with multiplanar reformations might facilitate lesion location by reducing partial volume effects. Unfortunately, in this study, readers had only access to 2D T2w axial and sagittal imaging.

As others [30], we found that experienced seniors performed significantly better, mostly because they assigned lower scores to non-csPCa lesions. However, the impact of experience on inter-reader agreement was small and agreement remained moderate at best, even for experienced seniors. This is discordant with another study in which inter-reader agreement was substantial and better between dedicated uro-radiologists than between non-dedicated radiologists. However, in that study, all radiologists were from the same institution, which may have reduced interpretation variability, particularly among dedicated radiologists [15]. Taken together, our results suggest that, despite continuous efforts of standardization and clarification, most PI-RADS descriptors remain subjective. Distinguishing ‘marked’ from ‘non-marked’ abnormalities, ‘encapsulated’ from ‘mostly encapsulated’ nodules, or ‘focal’ from ‘non-focal’ enhancement is subjective but has major effect on the final score. Interestingly, for PI-RADSv2.1 and PI-RADSv2, and for all groups of readers, *κ* values tended to be higher for T2-weighted and diffusion-weighted categories than for DCE categories. Although this finding should be interpreted with care since all pulse sequences do not have the same number of categories, it may suggest that visually distinguishing positive from negative cases is difficult at DCE, especially in the presence of subtle enhancements from background.

Several solutions for improving MRI reproducibility can be suggested. Mentoring through systematic double reading with an experienced reader could probably accelerate the training of beginners, but this is made difficult by the heavy workload of radiologists [31]. Using quantitative thresholds for apparent diffusion coefficient or DCE-derived parameters may also improve prostate MRI accuracy and inter-reader agreement [16, 32–34], but there is still progress to be made on the reproducibility of MRI biomarkers [35–38]. Finally, assistance by Artificial Intelligence algorithms may facilitate prostate MRI reading in the future; however, conflicting results have been recently published on this matter [39–45].

Our sample size was not designed to statistically compare PI-RADSv2.1 and PI-RADSv2 performances, because the difference was expected to be small. Meaningful comparison would have needed an unrealistic number of patients. Yet, the strict application of PI-RADSv2.1 descriptors in predefined lesions tended to yield lower scores in non-csPCa lesions as compared to PI-RADSv2 descriptors. This was mainly observed in PZ lesions for which the PI-RADSv2.1 clarifications on Dw imaging categories 2, 3 and 4 seem to have favoured better characterization. However, this effect was too small and too heterogeneous across readers to induce a substantial difference between the AUCs of the two scores. Additionally, PI-RADSv2.1 clarifications did not improve inter-reader agreement.

After assessing the predefined lesions, readers were allowed to describe additional suspicious lesions. This was designed to evaluate whether the new PI-RADSv2.1 upgrading rules in TZ increased the number of suspicious lesions as compared to PI-RADSv2. In accordance with other studies [12–14, 18], we found that such upgradings were rare. As a result, per-lobe analysis, that included predefined and additional lesions, showed similar results than per-lesion analysis: experienced seniors out-performed the two other groups of readers, and, in all groups of readers, PI-RADSv2.1 showed a trend toward improved specificity as compared to PI-RADSv2. Of note, the number of additional lesions was highly variable across readers, with juniors tending to describe more lesions that seniors.

In this study, experienced readers were a priori defined as having more than 5 years of experience. A recent European consensus suggested that a minimum of 1000 cases should be read to become an expert [31]. All our experienced seniors fulfilled that condition, and our results are in line with those of the European consensus. 

Readers assessed PI-RADSv2 and PI-RADSv2.1 during the same session. This may have resulted in underestimating the differences between the scores. However, independent scoring is illusory; most readers were familiar with the PI-RADSv2 descriptors and would have kept them in mind when using the new PI-RADSv2.1 criteria. In addition, assigning the scores in two different sessions introduces intra-reader variability, which may be substantial [46, 47]. Because reading the cases needed approximately 15–20 h, we were also afraid that the second reading would be biased by fatigue and the gradual lack of involvement of the readers. Thus, we chose to ask the readers to concentrate, during the same reading session, on the assessment of each pulse sequence category by following as closely as possible the written PI-RADSv2 and PI-RADSv2.1 descriptors without minding the overall score that was calculated automatically.

Our study has limitations. Firstly, because we indicated the predefined lesions to the readers, the AUCs obtained herein do not fully assess the diagnostic performance of the PI-RADS score in clinical routine. The detection phase, that is also a source of interpretation variability, was outside the scope of this study. However, many other studies have already assessed the overall performance of the PI-RADS score [23–25]. Instead, we wanted to specifically evaluate whether the PI-RADS descriptors were specific enough to induce reproducible characterization of the same lesion across multiple readers. This allowed the evaluation of factors of variability (size, location, PI-RADS categories of each pulse sequence) that, to our knowledge, had not been studied before. Secondly, prostate biopsy, used as reference standard, may have missed some csPCas. However, the small proportion of aggressive cancers detected during follow-up suggests that the sensitivity of our biopsy technique was good. Thirdly, we included only biopsy-naïve patients. Our results may not be valid for other populations.

In conclusion, when assessing the same set of MRI lesions using PI-RADSv2.1 and PI-RADSv2 descriptors, experienced seniors performed significantly better in characterizing csPCa than the other groups of readers. PI-RADSv2.1 descriptors tended to be more specific than PI-RADSv2 descriptors, but did not improve inter-reader variability.

## Supplementary Information

**Additional file 1.** Online Appendix.

## Data Availability

The biopsy databases was collected at The Hospices Civils de Lyon and is not publicly available. Pseudonymized data from MULTI dataset (i.e. individual score sheets of the readers) may be available from the corresponding author upon reasonable request. To gain access, data requestors will need to sign a data access agreement.

## Abbreviations

AUC: Area under the curve
CI: Confidence interval
csPCa: Clinically significant prostate cancer
CZ: Central zone
DCE: Dynamic contrast-enhanced
Dw: Diffusion-weighted
IQR: Interquartile range
ISUP: International society of urological pathology
MR: Magnetic resonance
MRI: Magnetic resonance imaging
PI-RADSv2.1: Prostate imaging-reporting and data system version 2.1
PI-RADSv2: Prostate imaging-reporting and data system version 2
PZ: Peripheral zone
ROC: Receiver operating characteristic
T2w: T2-weighted
TZ: Transition zone

## References

[1] Richenberg J, Logager V, Panebianco V, Rouviere O, Villeirs G, Schoots IG (2019). The primacy of multiparametric MRI in men with suspected prostate cancer. Eur Radiol.

[2] Drost FH, Osses DF, Nieboer D et al (2019) Prostate MRI, with or without MRI-targeted biopsy, and systematic biopsy for detecting prostate cancer. Cochrane Database Syst Rev 4: CD012663 10.1002/14651858.CD012663.pub2 PMC6483565 31022301

[3] Westphalen AC, McCulloch CE, Anaokar JM (2020). Variability of the positive predictive value of PI-RADS for prostate MRI across 26 centers: experience of the society of abdominal radiology prostate cancer disease-focused panel. Radiology.

[4] Greer MD, Shih JH, Lay N (2019). Interreader variability of prostate imaging reporting and data system version 2 in detecting and assessing prostate cancer lesions at prostate MRI. AJR Am J Roentgenol.

[5] Mussi TC, Yamauchi FI, Tridente CF (2020). Interobserver agreement of PI-RADS v. 2 lexicon among radiologists with different levels of experience. J Magn Reson Imaging.

[6] Barkovich EJ, Shankar PR, Westphalen AC (2019). A systematic review of the existing prostate imaging reporting and data system version 2 (PI-RADSv2) literature and subset meta-analysis of PI-RADSv2 categories stratified by gleason scores. AJR Am J Roentgenol.

[7] Park KJ, Choi SH, Lee JS, Kim JK, Kim MH, Jeong IG (2020). Risk stratification of prostate cancer according to PI-RADS(R) version 2 categories: meta-analysis for prospective studies. J Urol.

[8] Park KJ, Choi SH, Lee JS, Kim JK, Kim MH (2020). Interreader agreement with prostate imaging reporting and data system version 2 for prostate cancer detection: a systematic review and meta-analysis. J Urol.

[9] Rudolph MM, Baur ADJ, Haas M (2020). Validation of the PI-RADS language: predictive values of PI-RADS lexicon descriptors for detection of prostate cancer. Eur Radiol.

[10] Turkbey B, Rosenkrantz AB, Haider MA (2019). Prostate imaging reporting and data system version 2.1: 2019 update of prostate imaging reporting and data system version 2. Eur Urol.

[11] Tamada T, Kido A, Takeuchi M (2019). Comparison of PI-RADS version 2 and PI-RADS version 2.1 for the detection of transition zone prostate cancer. Eur J Radiol.

[12] Byun J, Park KJ, Kim MH, Kim JK (2020). Direct comparison of PI-RADS version 2 and 2.1 in transition zone lesions for detection of prostate cancer: preliminary experience. J Magn Reson Imaging.

[13] Lim CS, Abreu-Gomez J, Carrion I, Schieda N (2021). Prevalence of prostate cancer in PI-RADS version 2.1 transition zone atypical nodules upgraded by abnormal DWI: correlation With MRI-directed TRUS-guided targeted biopsy. AJR Am J Roentgenol.

[14] Costa DN, Jia L, Subramanian N (2021). Prospective PI-RADS v2.1 atypical benign prostatic hyperplasia nodules with marked restricted diffusion: detection of clinically significant prostate cancer on multiparametric MRI. AJR Am J Roentgenol.

[15] Brembilla G, Dell'Oglio P, Stabile A (2020). Interreader variability in prostate MRI reporting using prostate imaging reporting and data system version 2.1. Eur Radiol.

[16] Linhares Moreira AS, De Visschere P, Van Praet C, Villeirs G (2021). How does PI-RADS v2.1 impact patient classification? A head-to-head comparison between PI-RADS v2.0 and v2.1. Acta Radiol.

[17] Hotker AM, Bluthgen C, Rupp NJ, Schneider AF, Eberli D, Donati OF (2020). Comparison of the PI-RADS 2.1 scoring system to PI-RADS 2.0: Impact on diagnostic accuracy and inter-reader agreement. PLoS One.

[18] Rudolph MM, Baur ADJ, Cash H (2020). Diagnostic performance of PI-RADS version 21 compared to version 20 for detection of peripheral and transition zone prostate cancer. Sci Rep.

[19] Walker SM, Mehralivand S, Harmon SA (2020). Prospective evaluation of PI-RADS version 21 for prostate cancer detection. AJR Am J Roentgenol.

[20] Bhayana R, O'Shea A, Anderson MA (2021). PI-RADS versions 2 and 2.1: interobserver agreement and diagnostic performance in peripheral and transition zone lesions among six radiologists. AJR Am J Roentgenol.

[21] Xu L, Zhang G, Zhang D (2020). Comparison of PI-RADS version 2.1 and PI-RADS version 2 regarding interreader variability and diagnostic accuracy for transition zone prostate cancer. Abdom Radiol (NY).

[22] Wei CG, Zhang YY, Pan P (2021). Diagnostic accuracy and interobserver agreement of PI-RADS version 2 and version 2.1 for the detection of transition zone prostate cancers. AJR Am J Roentgenol.

[23] Lee CH, Vellayappan B, Tan CH (2022). Comparison of diagnostic performance and inter-reader agreement between PI-RADS v2.1 and PI-RADS v2: systematic review and meta-analysis. Br J Radiol.

[24] Park KJ, Choi SH, Kim MH, Kim JK, Jeong IG (2021). Performance of prostate imaging reporting and data system version 2.1 for diagnosis of prostate cancer: a systematic review and meta-analysis. J Magn Reson Imaging.

[25] Annamalai A, Fustok JN, Beltran-Perez J, Rashad AT, Krane LS, Triche BL (2022). Interobserver agreement and accuracy in interpreting mpMRI of the prostate: a systematic review. Curr Urol Rep.

[26] Habchi H, Bratan F, Paye A (2014). Value of prostate multiparametric magnetic resonance imaging for predicting biopsy results in first or repeat biopsy. Clin Radiol.

[27] Alonzo TA, Pepe MS (2002). Distribution-free ROC analysis using binary regression techniques. Biostatistics.

[28] Pepe MS (2003). The statistical evaluation of medical tests for classification and prediction.

[29] Mottet N, Bellmunt J, Bolla M (2017). EAU-ESTRO-SIOG guidelines on prostate cancer. part 1: screening, diagnosis, and local treatment with curative intent. Eur Urol.

[30] Stabile A, Giganti F, Kasivisvanathan V (2020). Factors influencing variability in the performance of multiparametric magnetic resonance imaging in detecting clinically significant prostate cancer: a systematic literature review. Eur Urol Oncol.

[31] de Rooij M, Israel B, Tummers M (2020). ESUR/ESUI consensus statements on multi-parametric MRI for the detection of clinically significant prostate cancer: quality requirements for image acquisition, interpretation and radiologists' training. Eur Radiol.

[32] Ullrich T, Schimmoller L (2020). Perspective: a critical assessment of PI-RADS 2.1. Abdom Radiol (NY).

[33] Moraes MO, Roman DHH, Copetti J (2020). Effects of the addition of quantitative apparent diffusion coefficient data on the diagnostic performance of the PI-RADS v2 scoring system to detect clinically significant prostate cancer. World J Urol.

[34] Abreu-Gomez J, Walker D, Alotaibi T, McInnes MDF, Flood TA, Schieda N (2020). Effect of observation size and apparent diffusion coefficient (ADC) value in PI-RADS v2.1 assessment category 4 and 5 observations compared to adverse pathological outcomes. Eur Radiol.

[35] Fedeli L, Belli G, Ciccarone A (2018). Dependence of apparent diffusion coefficient measurement on diffusion gradient direction and spatial position - a quality assurance intercomparison study of forty-four scanners for quantitative diffusion-weighted imaging. Phys Med.

[36] Shukla-Dave A, Obuchowski NA, Chenevert TL (2019). Quantitative imaging biomarkers alliance (QIBA) recommendations for improved precision of DWI and DCE-MRI derived biomarkers in multicenter oncology trials. J Magn Reson Imaging.

[37] Brunelle S, Zemmour C, Bratan F (2018). Variability induced by the MR imager in dynamic contrast-enhanced imaging of the prostate. Diagn Interv Imaging.

[38] Hoang-Dinh A, Nguyen-Quang T, Bui-Van L, Gonindard-Melodelima C, Souchon R, Rouviere O (2022). Reproducibility of apparent diffusion coefficient measurement in normal prostate peripheral zone at 1.5T MRI. Diagn Interv Imaging.

[39] Penzkofer T, Padhani AR, Turkbey B (2021). ESUR/ESUI position paper: developing artificial intelligence for precision diagnosis of prostate cancer using magnetic resonance imaging. Eur Radiol.

[40] Gaur S, Lay N, Harmon SA (2018). Can computer-aided diagnosis assist in the identification of prostate cancer on prostate MRI? a multi-center, multi-reader investigation. Oncotarget.

[41] Mehralivand S, Harmon SA, Shih JH (2020). Multicenter multireader evaluation of an artificial intelligence-based attention mapping system for the detection of prostate cancer with multiparametric MRI. AJR Am J Roentgenol.

[42] Zhu L, Gao G, Liu Y (2020). Feasibility of integrating computer-aided diagnosis with structured reports of prostate multiparametric MRI. Clin Imaging.

[43] Zhang KS, Schelb P, Netzer N (2022). Pseudoprospective paraclinical interaction of radiology residents with a deep learning system for prostate cancer detection: experience, performance, and identification of the need for intermittent recalibration. Invest Radiol.

[44] Labus S, Altmann MM, Huisman H (2023). A concurrent, deep learning-based computer-aided detection system for prostate multiparametric MRI: a performance study involving experienced and less-experienced radiologists. Eur Radiol.

[45] Rouviere O, Jaouen T, Baseilhac P (2022). Artificial intelligence algorithms aimed at characterizing or detecting prostate cancer on MRI: How accurate are they when tested on independent cohorts? - a systematic review. Diagn Interv Imaging.

[46] Niaf E, Lartizien C, Bratan F (2014). Prostate focal peripheral zone lesions: characterization at multiparametric MR imaging–influence of a computer-aided diagnosis system. Radiology.

[47] Smith CP, Harmon SA, Barrett T (2019). Intra- and interreader reproducibility of PI-RADSv2: a multireader study. J Magn Reson Imaging.

