# Supplementary material for: Characterization of high-grade prostate cancer at multiparametric MRI: assessment of PI-RADS version 2.1 and version 2 descriptors across 21 readers with varying experience (MULTI study)
Source: Insights Imaging. 2023 Mar 20;14:49. doi: 10.1186/s13244-023-01391-z (PMC10027981; doi:10.1186/s13244-023-01391-z)
Supplement: Supplementary file 1 — Additional file 1. Online Appendix. [file 13244_2023_1391_MOESM1_ESM.docx]

**Online Appendix**

**I. MULTI study group**

**I.1. Collaborators that participated to the MULTI study.**

**- Hospices Civils de Lyon**

**-** Department of Imaging, Hôpital Edouard Herriot: Stéphanie Bravetti, Stéphane Cadot, Bénédicte Cayot, Sabine Debeer, Florian Di Franco, Marine Dubreuil-Chambardel, Paul-Hugo Jouve de Guibert, Laurent Milot, Paul Cezar Moldovan, Gaele Pagnoux, Clément Pernet, Louis Perrier, Olivier Rouvière, Nicolas Stacoffe, Sarah Transin.

- Department of Urology, Hôpital Edouard Herriot: Marc Colombel, Sébastien Crouzet.

- Department of Imaging, Centre Hospitalier Lyon Sud: Domitille Cadiot, Leangsing Iv.

- Department of Urology, Centre Hospitalier Lyon Sud: Alain Ruffion.

- Department of Biostatistics: Mathilde Almeras, Amna Klich, Muriel Rabilloud,

**- Hôpital Saint Joseph Saint Luc, Lyon, France**

**-** Department of Radiology: Flavie Bratan, Rémy Rosset

**- University Hospital of Saint-Etienne, Hôpital Nord, Saint-Etienne, France**

**-** Department of Radiology: Athivada Soto Thammavong,

**- University Hospital of Dijon, Hôpital François-Mitterrand, Dijon, France:**

**-** Department of Vascular and Interventional Radiology, Image-Guided Therapy Center: Olivier Lopez

**- Imagerie médicale Val d’Ouest Charcot (IMVOC), Ecully, France:** Michel Abihanna, Sébastien Ronze.

**- Norimagerie, Caluire et Cuire, France:** Nicolas Girouin

**- Clinique de la Sauvegarde, Lyon, France:**

**-** Department of Radiology: Alexandre Ben Cheikh

**- Médipôle Lyon Villeurbanne, Villeurbanne, France**

**-** Department of Radiology : Jean Champagnac

**I. 2. Readers’ characteristics**

|  | Reader | Hospital | Type of institution | Experience (Years) | Nb of unsupervised cases read | |
| --- | --- | --- | --- | --- | --- | --- |
| Experienced seniors | 1 | 1 | Private hospital | 15 | ≥1500 | |
|  | 2 | 2 | Private hospital | 15 | ≥1500 | |
|  | 3 | 3 | Private hospital | 15 | 1000-1500 | |
|  | 4 | 4 | Public non-academic hospital | 10 | ≥1500 | |
|  | 5 | 5 | Public academic hospital | 9 | ≥1500 | |
|  | 6 | 6 | Public academic hospital | 5 | 1000-1500 | |
|  | 7 | 5 | Public academic hospital | 5 | 1000-1500 | |
| Less experienced seniors | 8 | 4 | Public non-academic hospital | 3 | 1000-1500 | |
|  | 9 | 5 | Public academic hospital | 4 | ≥1500 | |
|  | 10 | 7 | Public academic hospital | 3 | 1000-1500 | |
|  | 11 | 5 | Public academic hospital | 2 | 400-1000 | |
|  | 12 | 5 | Public academic hospital | 1 | <400 | |
|  | 13 | 8 | Public academic hospital | 1 | <400 | |
|  | 14 | 5 | Public academic hospital | 1 | <400 | |
|  | Reader | Institution | Type of institution | Year in training | Rotation in uro-radiology | Advanced diploma in uro-radiology |
| Juniors | 15 | 5 | Public academic hospital | 5th | Yes | Yes |
|  | 16 | 9 | Public academic hospital | 4th | No | Yes |
|  | 17 | 5 | Public academic hospital | 4th | Yes | Yes |
|  | 18 | 5 | Public academic hospital | 4th | Yes | No |
|  | 19 | 5 | Public academic hospital | 3rd | Yes | No |
|  | 20 | 5 | Public academic hospital | 5th | No | No |
|  | 21 | 5 | Public academic hospital | 3rd | No | No |

**I.3 Readers’ institutions**

The institutions of the readers were the following:

- **Institution 1:** Imagerie médicale Val d’Ouest Charcot (IMVOC), Ecully, France
- **Institution 2:** Norimagerie, Caluire et Cuire, France
- **Institution 3:** Department of Radiology, Clinique de la Sauvegarde, Lyon, France
- **Institution 4:** Department of Radiology, Hôpital Saint Joseph Saint Luc, Lyon, France
- **Institution 5:** Department of Radiology, Hospices Civils de Lyon, Hôpital Edouard Herriot, Lyon France
- **Institution 6:** Department of Radiology, Médipôle Lyon Villeurbanne, Villeurbanne, France
- **Institution 7:** Department of Radiology, University Hospital of Saint-Etienne, Hôpital Nord, Saint-Etienne, France
- **Institution 8:** Department of Radiology, Hospices Civils de Lyon, Centre Hospitalier Lyon Sud, Lyon, France
- **Institution 9:** Department of Vascular and Interventional Radiology, Image-Guided Therapy Center: University Hospital of Dijon, Hôpital François-Mitterrand, Dijon, France

**II. PI-RADS descriptors used by the readers**

The PI-RADS version 2 guidelines can be found in the following references:

- Weinreb JC, Barentsz JO, Choyke PL et al (2016), PI-RADS Prostate Imaging – Reporting and Data System: 2015, version 2. Eur Urol 69:16-40
- Barentsz JO, Weinreb JC, Verma S et al (2016), Synopsis of the PI-RADS v2 guidelines for multiparametric prostate magnetic resonance imaging and recommendations for use. Eur Urol 69:41-49

The PI-RADS version 2.1 guidelines can be found in the following references:

- Turkbey B, Rosenkrantz AB, Haider MA et al (2019), Prostate Imaging Reporting and Data System Version 2.1: 2019 Update of Prostate Imaging Reporting and Data System Version 2. Eur Urol 76:340-351
- PI-RADS 2019 version 2.1: https://www.acr.org/-/media/ACR/Files/RADS/Pi-RADS/PIRADS-v2-1.pdf

**II.1. PI-RADS v2 scoring**

**T2-weighted imaging categories**

|  | Peripheral Zone | Transition zone |
| --- | --- | --- |
| 1 | Uniform hyperintense signal intensity (normal) | Homogeneous intermediate signal intensity (normal) |
| 2 | Linear, wedge-shaped, or diffuse mild hypointensity, usually indistinct margin | Circumscribed hypointense or heterogeneous encapsulated nodule(s) (BPH) |
| 3 | Heterogeneous signal intensity or non-circumscribed, rounded, moderate hypointensity | Heterogeneous signal intensity with obscured margins. Includes others that do not qualify as 2, 4, or 5. |
| 4 | Circumscribed, homogeneous moderate hypointense focus/mass confined to prostate and <1.5 cm in greatest dimension | Lenticular or non-circumscribed, homogeneous, moderately hypointense, and < 1.5 cm in greatest dimension |
| 5 | Same as 4, but ≥1.5 cm in greatest dimension or definite extraprostatic extension/invasive behavior | Same as 4, but ≥1.5 cm in greatest dimension or definite extraprostatic extension/invasive behavior |

**Diffusion-weighted imaging categories**

|  | Peripheral zone and Transition zone |
| --- | --- |
| 1 | No abnormality (i.e. normal) on ADC and high b-value DWI |
| 2 | Indistinct hypointense on ADC |
| 3 | Focal mildly/moderately hypointense on ADC and isointense/mildly hyperintense on high b-value DWI |
| 4 | Focal markedly hypointense on ADC and markedly hyperintense on high b-value DWI  <1.5 cm on axial images |
| 5 | Same as 4, but ≥1.5 cm in greatest dimension or definite extraprostatic extension/invasive behavior |

**Dynamic contrast-enhanced imaging categories**

|  | Peripheral zone and Transition zone |
| --- | --- |
| Negative | No early enhancement,  OR diffuse enhancement not corresponding to a focal finding on T2W and/or DWI, or;  OR focal enhancement corresponding to a lesion demonstrating features of BPH on T2W |
| Positive | Focal,  AND earlier than or contemporaneously with enhancement of adjacent tissues and;  AND corresponds to suspicious findings on T2W and/or DWI |

**II.2. Score PI-RADS 2.1**

**T2-weighted imaging categories**

|  | Peripheral Zone | Transition zone |
| --- | --- | --- |
| 1 | Uniform hyperintense signal intensity (normal) | Normal appearing TZ (rare) or a round, completely encapsulated nodule (“typical nodule”) |
| 2 | Linear or wedge-shaped, or diffuse mild hypointensity, usually indistinct margin | A mostly encapsulated nodule  OR a homogeneous circumscribed nodule without encapsulation (“atypical nodule”)  OR a homogeneously mildly hypointense area between nodules |
| 3 | Heterogeneous signal intensity or non-circumscribed, rounded, moderate hypointensity  Includes others that do not qualify as 2, 4, or 5. | Heterogeneous signal intensity with obscured margins.  Includes others that do not qualify as 2, 4, or 5. |
| 4 | Circumscribed, homogeneous moderate hypointense focus/mass confined to prostate and <1.5 cm in greatest dimension | Lenticular or non-circumscribed, homogeneous, moderately hypointense, and < 1.5 cm in greatest dimension |
| 5 | Same as 4, but ≥1.5 cm in greatest dimension or definite extraprostatic extension/invasive behavior | Same as 4, but ≥1.5 cm in greatest dimension or definite extraprostatic extension/invasive behavior |

**Diffusion-weighted imaging categories**

|  | Peripheral zone and Transition zone |
| --- | --- |
| 1 | No abnormality (i.e. normal) on ADC and high b-value DWI |
| 2 | Linear/wedge shaped hypointense on ADC and/or linear/wedge shaped hyperintense on high b-value DWI |
| 3 | Focal (discrete and different from the background) hypointense on ADC and/or focal hyperintense on high b-value DWI; may be markedly hypointense on ADC or markedly hyperintense on high b-value DWI but not both |
| 4 | Focal markedly hypointense on ADC and markedly hyperintense on high b-value DWI  <1.5 cm on axial images |
| 5 | Same as 4, but ≥1.5 cm in greatest dimension or definite extraprostatic extension/invasive behavior |

**Dynamic contrast-enhanced imaging categories**

|  | Peripheral zone and Transition zone |
| --- | --- |
| Negative | No early or contemporaneous enhancement,  OR diffuse multifocal enhancement NOT corresponding to a focal finding on T2W and/or DWI,  OR focal enhancement corresponding to a lesion demonstrating features of BPH on T2W (including features of extruded BPH in the PZ) |
| Positive | Focal,  AND earlier than or contemporaneously with enhancement of adjacent tissues,  AND corresponds to suspicious findings on T2W and/or DWI |

**III. Principles of per-lobe and per-patient scoring**


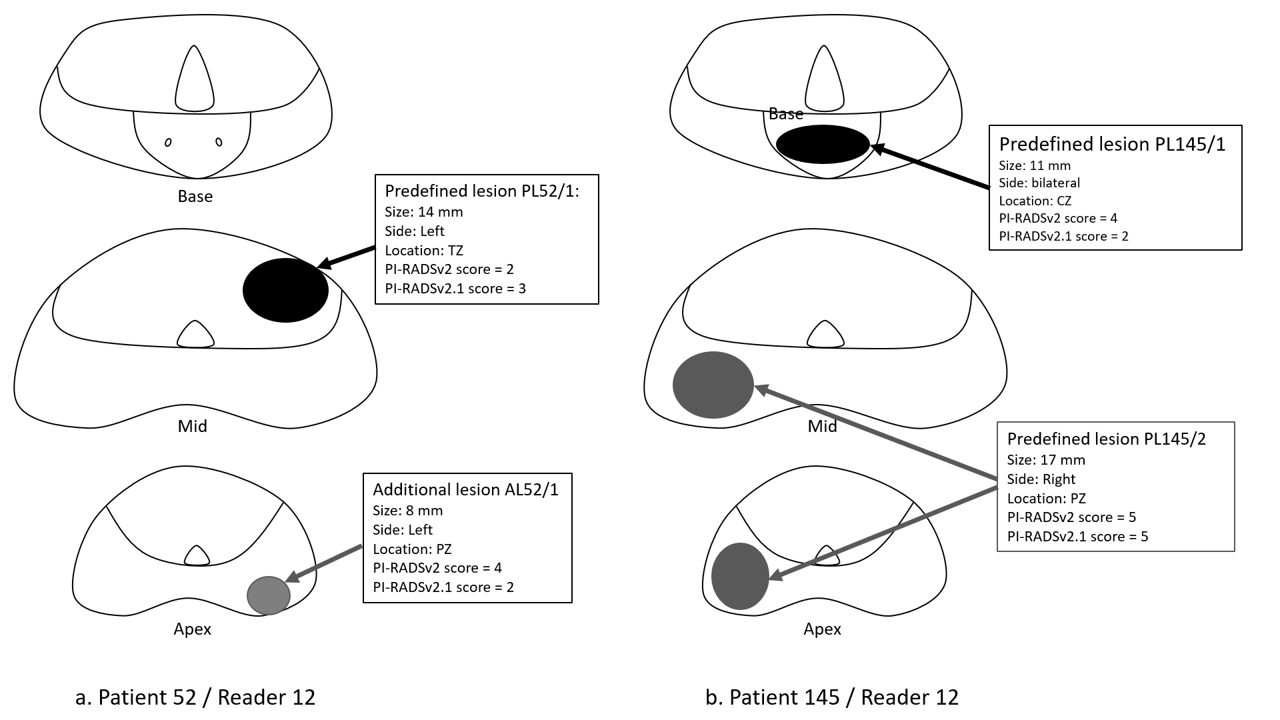


Schematic diagram showing the principles of per-lobe and per-patient scoring:

a. Patient 52 had one predefined lesion (PL52/1). Reader 12 considered that PL52/1 was in the left TZ, had a size of 14 mm, a PI-RADSv2 score of 2 and a PI-RADSv2.1 score of 3. She also described an additional lesion (AL52/1) in the left PZ at the apex, with a size of 8 mm, a PI-RADSv2 score of 4 and a PI-RADSv2.1 score of 2. The final PI-RADSv2 and PI-RADSv2.1 scores of the right lobe for this reader are 1 (no lesion). The final PI-RADSv2 sand PI-RADSv2.1 score of the left lobe are 4 and 3 respectively. Patient 52 final PI-RADSv2 sand PI-RADSv2.1 scores for this reader are 4 and 3 respectively.

b. Patient 145 had two predefined lesions (PL145/1 and PL145/2). Reader 12 considered that PL145/1 was bilateral, in the CZ, had a size of 11 mm, a PI-RADSv2 score of 4, and a PI-RADSv2.1 score of 2. She considered that PL145/2 was in the right PZ, had a size of 17 mm, a PI-RADSv2 score of 5, and a PI-RADSv2.1 score of 5. The final PI-RADSv2 and PI-RADSv2.1 scores of the right lobe for this reader are 5. The final PI-RADSv2 sand PI-RADSv2.1 score of the left lobe are 4 and 2, respectively. Patient 145 final PI-RADSv2 sand PI-RADSv2.1 scores for this reader are 5.

**IV. Prostate multiparametric magnetic prostate imaging parameters**

| Scanner | Nb of patients | Pulse sequence | TR (ms) | TE (ms) | FOV (cm) | Matrix | b-values (s/mm²) | Angle (°) | Slice thickness (mm) | Nb of temporal acquisitions | Temporal resolution (s) |
| --- | --- | --- | --- | --- | --- | --- | --- | --- | --- | --- | --- |
| General Electric, MR 750 (3T) | 63 | T2WI | 3994-5210 | 140-149 | 220-280 | 384x256 | - | 146 | 3 | - | - |
|  |  | DWI | 4500 | 69 | 380-400 | 128x128 | 0, 50, 150, 300, 800, 2000 | 90 | 3 | - | - |
|  |  | DCEI | 3.7-4.1 | 2 | 240-320 | 160x180 | - | 12 | 3 | 26-33 | 6.9-8.8 |
| General Electric, MR 450 (1.5T) | 70 | T2WI | 4977-7465 | 98 - 99 | 240-280 | 288x288 | - | 140 | 3 | - | - |
|  |  | DWI | 4288-6074 | 86 | 360x360 | 86x110 | 0, 50, 150, 800, 2000 | 90 | 3 | - | - |
|  |  | DCEI | 6.2-6.4 | 3 | 240-340 | 140x224 | - | 12 | 3 | 27-34 | 8.4-13.8 |
| Philips,  Ingenia (3T) | 19 | T2WI | 5000 | 110 | 230 | 356x251-356x269 | - | 90 | 3 | - | - |
|  |  | DWI | 8535-9576 | 64-70 | 285-321 | 106x108, 94x96 | 0, 50, 150, 300, 800, 1200, 2000 | 90 | 3 | - | - |
|  |  | DCEI | 4.8-5.5 | 1.5 | 275 | 212x216 | - | 10-12 | 6 | 40 | 6.6-7.6 |
| Philips,  Ingenia (1.5T) | 7 | T2WI | 6926 | 95 | 200 | 324x273 | - | 90 | 3 | - | - |
|  |  | DWI | 2148-2198 | 80-81 | 350 | 98x100-100x100, | 0, 2000 | 90 | 3 | - | - |
|  |  | DCEI | 7.4-7.5 | 4.2 | 285 | 192x190 | - | 20 | 6 | 25 | 11.8-11.9 |

Nb: number; TR: repetition time; TE: echo time; FOV: field of view; T2WI: T2-weighted imaging; DWI: diffusion-weighted imaging; DCEI: dynamic contrast-enhanced imaging

In the absence of contraindication, butylscopolamin or glucagon was injected at the start of the examination.

.

**V. Analysis of the 240 predefined lesions**

**V.1. Location and size of predefined lesions: individual results**

|  | **Reader** | **PZ lesions** | | **TZ lesions** | | **CZ lesions** | | **Median size of the 240 lesions (mm)** |
| --- | --- | --- | --- | --- | --- | --- | --- | --- |
|  |  | **Total Nb** | **csPCa** | **Total Nb** | **csPCa** | **Total Nb** | **csPCa** |  |
| Experienced seniors | 1 | 211 | 67 | 21 | 9 | 8 | 4 | 8 |
|  | 2 | 200 | 66 | 26 | 9 | 14 | 5 | 10 |
|  | 3 | 204 | 63 | 24 | 8 | 12 | 9 | 11 |
|  | 4 | 207 | 66 | 28 | 11 | 5 | 3 | 11 |
|  | 5 | 204 | 68 | 29 | 9 | 7 | 3 | 11.5 |
|  | 6 | 212 | 69 | 23 | 8 | 5 | 3 | 10 |
|  | 7 | 213 | 68 | 19 | 7 | 8 | 5 | 10 |
|  | Median [IQR] | 207 [204-212] | 67 [66-68] | 24 [22-27] | 9 [8-9] | 8 [6-10] | 4 [3-5] | 10 [10-11] |
| Less experienced seniors | 8 | 182 | 60 | 33 | 12 | 25 | 8 | 8 |
|  | 9 | 204 | 67 | 31 | 11 | 4 | 2 | 10 |
|  | 10 | 213 | 68 | 24 | 10 | 3 | 2 | 10 |
|  | 11 | 206 | 70 | 22 | 8 | 12 | 2 | 10 |
|  | 12 | 209 | 68 | 25 | 9 | 6 | 3 | 12 |
|  | 13 | 210 | 67 | 20 | 8 | 10 | 5 | 10 |
|  | 14 | 197 | 62 | 31 | 11 | 12 | 7 | 11 |
|  | Median [IQR] | 206 [201-210] | 67 [65-68] | 25 [23-31] | 10 [9-11] | 10 [5-12] | 3 [2-6] | 10 [10-11] |
| Juniors | 15 | 182 | 60 | 27 | 9 | 31 | 15 | 11 |
|  | 16 | 203 | 67 | 27 | 9 | 10 | 5 | 11 |
|  | 17 | 202 | 68 | 28 | 10 | 10 | 3 | 10 |
|  | 18 | 192 | 70 | 27 | 11 | 21 | 12 | 11 |
|  | 19 | 212 | 68 | 23 | 7 | 5 | 3 | 12 |
|  | 20 | 202 | 67 | 27 | 11 | 11 | 8 | 11 |
|  | 21 | 209 | 62 | 25 | 10 | 6 | 3 | 10 |
|  | Median [IQR] | 202 [197-206] | 67 [65-68] | 27 [26-27] | 10 [9-11] | 10 [8-16] | 5 [3-10] | 11 [10-11] |
| All readers | Median [IQR] | 204 [202-210] | 67 [66-68] | 29 [24-62] | 9 [8-11] | 10 [6-12] | 4 [3-7] | 10 [10-11] |

TZ: transition zone; CZ: central zone; Nb: number; csPCa: clinically significant prostate cancer; IQR: interquartile range.

**V.2. Detection rates of clinically significant prostate cancer as a function of PI-RADSv2.1 and v2 PI-RADSv2 final scores in the 240 predefined lesions**

| Reader | PI-RADSv2.1 | | | | | PI-RADSv2 | | | | |
| --- | --- | --- | --- | --- | --- | --- | --- | --- | --- | --- |
|  | 1 | 2 | 3 | 4 | 5 | 1 | 2 | 3 | 4 | 5 |
| 1 | 0/3  (0%) | 5/77 (6%) | 7/40 (18%) | 41/84 (49%) | 27/36 (75%) | 1/2 (50%) | 5/75 (7%) | 6/41 (15%) | 41/86 (48%) | 27/36 (75%) |
| 2 | 2/16 (13%) | 5/55 (9%) | 4/28 (14%) | 35/100 (35%) | 34/41 (83%) | 2/11 (18%) | 2/30 (7%) | 4/30 (13%) | 38/128 (30%) | 34/41 (83%) |
| 3 | 12/86 (14%) | 3/21 (14%) | 4/22 (18%) | 33/72 (46%) | 28/39 (72%) | 11/84 (13%) | 4/27 (15%) | 5/22 (23%) | 32/68 (47%) | 28/39 (72%) |
| 4 | 2/11 (18%) | 7/56 (13%) | 7/24 (29%) | 26/98 (27%) | 38/51 (75%) | 3/13 (23%) | 3/31 (10%) | 8/30 (27%) | 28/115 (24%) | 38/51 (75%) |
| 5 | 0/0  (-) | 2/17 (12%) | 5/34 (15%) | 35/135 (26%) | 38/54 (70%) | 0/0  (-) | 2/13 (15%) | 5/34 (15%) | 35/139 (25%) | 38/54 (70%) |
| 6 | 0/4  (0%) | 9/85 (11%) | 11/39 (28%) | 36/81 (44%) | 24/31 (77%) | 0/3  (0%) | 8/61 (13%) | 10/53 (19%) | 38/91 (42%) | 24/32 (75%) |
| 7 | 1/2 (50%) | 1/22 (5%) | 2/20 (10%) | 44/147 (30%) | 32/49 (65%) | 0/0  (-) | 1/11 (9%) | 1/20 (5%) | 46/160 (29%) | 32/49 (65%) |
| 8 | 1/5 (20%) | 13/54 (24%) | 6/21 (29%) | 37/126 (29%) | 23/34 (68%) | 0/3  (0%) | 4/27 (15%) | 4/17 (24%) | 48/158 (30%) | 24/35 (69%) |
| 9 | 0/0  (-) | 1/19 (5%) | 4/23 (17%) | 40/150 (27%) | 35/48 (73%) | 0/0  (-) | 3/27 (11%) | 2/20 (10%) | 40/145 (28%) | 35/48 (73%) |
| 10 | 0/0  (-) | 0/21 (0%) | 14/77 (18%) | 39/106 (37%) | 27/36 (75%) | 0/0  (-) | 0/24 (0%) | 13/74 (18%) | 38/105 (36%) | 29/37 (78%) |
| 11 | 2/11 (18%) | 0/9  (0%) | 4/15 (27%) | 41/152 (27%) | 33/53 (62%) | 1/6 (17%) | 0/10 (0%) | 4/15 (27%) | 41/155 (27%) | 34/54 (63%) |
| 12 | 0/5  (0%) | 0/20 (0%) | 1/17 (6%) | 38/128 (30%) | 41/70 (59%) | 0/2  (0%) | 0/14 (0%) | 1/18 (6%) | 38/136 (28%) | 41/70 (59%) |
| 13 | 1/2 (50%) | 0/4  (0%) | 2/11 (18%) | 44/171 (26%) | 33/52 (64%) | 0/1  (0%) | 1/5 (20%) | 2/10 (10%) | 44/173 (26%) | 33/51 (65%) |
| 14 | 1/5 (20%) | 3/41 (7%) | 6/18 (33%) | 36/132 (27%) | 34/44 (77%) | 1/4 (25%) | 6/35 (17%) | 3/19 (16%) | 36/138 (26%) | 34/44 (77%) |
| 15 | 0/0  (-) | 0/1  (0%) | 2/26 (8%) | 45/151 (30%) | 33/62 (53%) | 0/0  (-) | 0/4  (0%) | 2/23 (9%) | 45/151 (30%) | 33/62 (53%) |
| 16 | 0/2  (0%) | 4/26 (15%) | 15/58 (26%) | 35/120 (29%) | 26/34 (76%) | 0/1  (0%) | 1/26 (4%) | 11/49 (22%) | 41/128 (32%) | 27/36 (75%) |
| 17 | 1/9 (11%) | 2/20 (10%) | 2/12 (17%) | 39/144 (27%) | 36/55 (65%) | 0/2  (0%) | 1/14 (7%) | 2/20 (10%) | 41/149 (28%) | 36/55 (65%) |
| 18 | 1/9 (11%) | 11/77 (14%) | 10/44 (23%) | 26/66 (39%) | 32/44 (73%) | 1/7 (14%) | 3/24 (13%) | 16/80 (20%) | 28/85 (33%) | 32/44 (73%) |
| 19 | 1/10 (10%) | 1/21 (5%) | 4/35 (11%) | 33/104 (32%) | 41/70 (59%) | 1/9 (11%) | 2/13 (15%) | 3/40 (8%) | 33/108 (31%) | 41/70 (59%) |
| 20 | 1/10 (10%) | 15/84 (18%) | 8/41 (20%) | 35/76 (46%) | 21/29 (72%) | 0/7 (0%) | 7/45 (16%) | 10/51 (20%) | 42/108 (39%) | 21/29 (72%) |
| 21 | 0/1  (0%) | 6/44 (14%) | 11/44 (25%) | 38/111 (34%) | 25/40 (63%) | 0/0  (-) | 3/24 (13%) | 12/58 (21%) | 40/118 (34%) | 25/40 (63%) |

**V.3. Areas under the receiver operating characteristic curves obtained by the readers using PI-RADSv2 and PI-RADSv2.1 in the 240 predefined lesions**

|  | **Reader** | **PI-RADS v2.1** | **PI-RADS v2** |
| --- | --- | --- | --- |
| Experienced seniors | 1 | 0.83 [0.79-0.88] | 0.79 [0.74-0.87] |
|  | 2 | 0.81 [0.77-0.86] | 0.80 [0.75-8-0.86] |
|  | 3 | 0.78 [0.72-0.84] | 0.78 [0.73-0.84] |
|  | 4 | 0.77 [0.72-0.82] | 0.76 [0.71-0.81] |
|  | 5 | 0.77 [0.70-0.83] | 0.76 [0.70-0.82] |
|  | 6 | 0.80 [0.75-0.85] | 0.77 [0.72-0.82] |
|  | 7 | 0.74 [0.68-0.81] | 0.75 [0.68-0.81] |
|  | Mean | 0.80 [0.76-0.84] | 0.79 [0.75-0.84] |
| Less experienced seniors | 8 | 0.66 [0.60-0.73] | 0.71 [0.66-0.77] |
|  | 9 | 0.78 [0.73-0.83] | 0.78 [0.72-0.83] |
|  | 10 | 0.77 [0.71-0.83] | 0.79 [0.73-0.85] |
|  | 11 | 0.71 [0.65-0.77] | 0.72 [0.66-0.77] |
|  | 12 | 0.78 [0.70-0.81] | 0.77 [0.69-0.81] |
|  | 13 | 0.71 [0.65-0.76] | 0.72 [0.66-0.77] |
|  | 14 | 0.78 [0.73-0.83] | 0.75 [0.70-0.81] |
|  | Mean | 0.74 [0.70-0.78] | 0.75 [0.70-0.79] |
| Juniors | 15 | 0.70 [0.64-0.76] | 0.70 [0.64-0.76] |
|  | 16 | 0.72 [0.66-0.79] | 0.77 [0.71-0.81] |
|  | 17 | 0.75 [0.69-0.81] | 0.77 [0.711-0.82] |
|  | 18 | 0.77 [0.72-0.83] | 0.75 [0.69-0.81] |
|  | 19 | 0.76 [0.70-0.81] | 0.75 [0.68-0.80] |
|  | 20 | 0.74 [0.69-0.80] | 0.74 [0.68-0.80] |
|  | 21 | 0.70 [0.65-0.77] | 0.70 [0.64-0.76] |
|  | Mean | 0.74 [0.70-0.79] | 0.75 [0.70-0.79] |

**V.4. Sensitivities and specificities obtained by the readers using PI-RADSv2.1 in the 240 lesions**

|  | Reader | Threshold ≥3 | | Threshold ≥4 | |
| --- | --- | --- | --- | --- | --- |
|  |  | Se [95% CI] | Spe [95% CI] | Se [95% CI] | Spe [95% CI] |
| Experienced seniors | 1 | 94 [86-98] | 47 [39-55] | 85 [75.3-92] | 68 [60-75] |
|  | 2 | 91 [83-96] | 40 [32-48] | 86 [77-93] | 55 [47-63] |
|  | 3 | 81 [71-89] | 58 [49-65] | 76 [65-85] | 69 [61-76] |
|  | 4 | 89 [80-95] | 36 [29-44] | 80 [70-88] | 47 [39-55] |
|  | 5 | 98 [91-100] | 9 [5-15] | 91 [83-96] | 28 [21-35] |
|  | 6 | 89 [80-95] | 50 [42-58] | 75 [64-84] | 68 [60-75] |
|  | 7 | 98 [91-100] | 14 [9-20] | 95 [88-99] | 25 [19-32] |
|  | Mean | 93 [85-97] | 33 [19-52] | 85 [76-92] | 51 [36-65] |
| Less experienced seniors | 8 | 83 [73-90] | 28 [21-36] | 75 [64-84] | 38 [30-45] |
|  | 9 | 99 [93-100] | 11 [7-17] | 94 [86-98] | 23 [17-30] |
|  | 10 | 100 [96-100] | 13 [8-19] | 83 [72-90] | 53 [44-60] |
|  | 11 | 98 [91-100] | 11 [7-17] | 93 [84-97] | 18 [12-25] |
|  | 12 | 100 [96-100] | 16 [10-22] | 99 [93-100] | 26 [19-33] |
|  | 13 | 99 [93-100] | 3 [1-7] | 96 [89-99] | 9 [5-14] |
|  | 14 | 95 [88-99] | 26 [20-34] | 88 [78-94] | 34 [26-42] |
|  | Mean | 98 [95-99] | 13 [7-25] | 91 [85-95] | 26 [16-40] |
| Juniors | 15 | 100 [96-100] | 1 [0-3] | 98 [91-100] | 16 [10-22] |
|  | 16 | 95 [88-99] | 15 [10-21] | 76 [65-85] | 42 [34-50] |
|  | 17 | 96 [89-99] | 16 [11-23] | 94 [86-98] | 23 [16-30] |
|  | 18 | 85 [75-92] | 46 [38-54] | 73 [61-82] | 68 [60-75] |
|  | 19 | 98 [91-100] | 18 [12-25] | 93 [84-97] | 38 [30-46] |
|  | 20 | 80 [70-88] | 49 [41-57] | 70 [59-80] | 69 [62-76] |
|  | 21 | 93 [84-97] | 24 [18-32] | 79 [68-87] | 45 [37-53] |
|  | Mean | 94 [88-98] | 19 [10-33] | 86 [76-92] | 42 [28-56] |

Sensitivities and specificities are expressed in percentages.

Se: sensitivity; Spe: specificity. 95% CI: 95% confidence interval.

**V.5. Sensitivities and specificities obtained by the readers using PI-RADSv2 in the 240 lesions**

|  | Reader | Threshold ≥3 | | Threshold ≥4 | |
| --- | --- | --- | --- | --- | --- |
|  |  | Se [95% CI] | Spe [95% CI] | Se [95% CI] | Spe [95% CI] |
| Experienced seniors | 1 | 93 [84-97] | 44 [37-52] | 85 [75-92] | 66 [58-74] |
|  | 2 | 95 [88-99] | 23 [17-30] | 90 [81-96] | 39 [32-47] |
|  | 3 | 81 [71-89] | 60 [52-68] | 75 [64-84] | 71 [63-78] |
|  | 4 | 93 [84-97] | 24 [17-31] | 83 [72-90] | 38 [30-45] |
|  | 5 | 98 [91-100] | 7 [3-12] | 91 [83-96] | 25 [19-32] |
|  | 6 | 90 [81-96] | 35 [28-43] | 78 [67-86] | 62 [54-69] |
|  | 7 | 99 [93-100] | 6 [3-11] | 98 [91-100] | 18 [12-25] |
|  | Mean | 94 [89-97] | 24 [15-38] | 87 [79-92] | 45 [31-59] |
| Less experienced seniors | 8 | 95 [88-99] | 16 [11-23] | 90 [81-96] | 24 [18-32] |
|  | 9 | 96 [89-99] | 15 [10-21] | 94 [86-98] | 26 [20-34] |
|  | 10 | 100 [95-100] | 15 [10-21] | 84 [74-91] | 53 [45-61] |
|  | 11 | 99 [93-100] | 9 [5-15] | 94 [86-98] | 16 [11-23] |
|  | 12 | 100 [95-100] | 10 [6-16] | 99 [93-100] | 21 [15-28] |
|  | 13 | 99 [93-100] | 3 [1-7] | 96 [89-99] | 8 [4-13] |
|  | 14 | 91 [83-96] | 20 [14-27] | 88 [78-94] | 30 [23-38] |
|  | Mean | 98 [95-99] | 11 [6-20] | 93 [88-96] | 24 [15-36] |
| Juniors | 15 | 100 [95.5-100] | 3 [1-6] | 98 [91-100] | 16 [10-22] |
|  | 16 | 99 [93-100] | 16 [11-23] | 85 [75-92] | 40 [32-48] |
|  | 17 | 99 [93-100] | 9 [5-15] | 96 [89-99] | 20.6 [15-28] |
|  | 18 | 95 [88-99] | 17 [11-24] | 75 [64-84] | 57[49-65] |
|  | 19 | 96 [89-99] | 12 [7-18] | 93 [84-97] | 35 [28-43] |
|  | 20 | 91 [83-96] | 28 [21-36] | 79 [68-87] | 54 [46-62] |
|  | 21 | 96 [89-99] | 13 [8-19] | 81 [71-89] | 42 [34-50] |
|  | Mean | 97 [95-99] | 12 [7-21] | 88 [81-93] | 36 [24-50] |

Sensitivities and specificities are expressed in percentages.

Se: sensitivity; Spe: specificity. 95% CI: 95% confidence interval.

**V.6. Downgrading and upgrading combinations obtained with PI-RADSv2.1 as compared to PI-RADSv2 in the 240 predefined lesions**

|  | Reader | Downgrading combinations from PI-RADSv2 to PI-RADSv2.1 | | | | | | | | | | | Upgrading combinations from PI-RADSv2 to PI-RADSv2.1 | | | | | | | |
| --- | --- | --- | --- | --- | --- | --- | --- | --- | --- | --- | --- | --- | --- | --- | --- | --- | --- | --- | --- | --- |
|  |  | 2 to 1 | 3 to 1 | 4 to 1 | 5 to 1 | 3 to 1 | 3 to 2 | 4 to 2 | 5 to 2 | 4 to 3 | 5 to 3 | 5 to 4 | 1 to 2 | 1 to 3 | 1 to 4 | 2 to 3 | 2 to 4 | 2 to 5 | 3 to 4 | 4 to 5 |
| Experienced seniors | 1 |  | 1 | 1 |  |  | 3 | 1(1) |  | 1 |  |  |  |  | 1(1) | 2(1) |  |  |  |  |
|  | 2 | 3 |  | 3 |  |  | 8(2) | 30(2) |  | 2(2) |  |  | 1 |  |  | 4 | 7(1) |  |  |  |
|  | 3 | 2(1) |  |  |  |  | 2(1) |  |  |  |  |  |  |  |  | 2 | 4(1) |  |  |  |
|  | 4 |  |  |  |  |  | 9(1) | 15(2) |  | 3 |  |  | 2(1) |  |  | 1 |  |  | 1 |  |
|  | 5 |  |  |  |  |  | 1 | 4 |  | 2 |  |  |  |  |  |  | 1 |  | 1 |  |
|  | 6 |  | 1 |  |  |  | 20(1) | 11(2) |  |  | 1 |  |  |  |  | 6(2) | 1 |  |  |  |
|  | 7 | 2(1) |  |  |  |  | 6 | 8(1) |  | 6(1) |  |  |  |  |  | 1 |  |  | 1 |  |
| Less experienced seniors | 8 | 2(1) |  |  |  |  | 5(1) | 33(10) | 1(1) | 3(1) |  |  |  |  |  | 6(2) | 4 |  |  |  |
|  | 9 |  |  |  |  |  |  |  |  |  |  |  |  |  |  | 3(2) | 5 |  |  |  |
|  | 10 |  |  |  |  |  | 2 |  |  | 1 | 1(1) | 1 |  |  |  | 3 | 2 |  |  | 1 |
|  | 11 | 1 |  | 4(1) | 1(1) |  |  |  |  |  |  |  |  |  | 1(1) |  |  |  |  |  |
|  | 12 | 3 |  |  |  |  | 3 | 8 |  | 1 |  |  |  |  |  | 1 | 1 |  |  |  |
|  | 13 | 1(1) |  |  |  |  |  | 2 |  |  |  |  |  |  |  | 1 |  | 1 |  |  |
|  | 14 | 1 |  |  |  |  | 8 | 8 |  | 4(1) |  |  |  |  |  | 3(2) | 6(1) |  |  |  |
| Juniors | 15 |  |  |  |  |  |  |  |  |  |  |  |  |  |  | 3 |  |  |  |  |
|  | 16 |  |  |  |  | 1 | 7(1) | 7(3) |  | 8(3) | 1 | 1(1) |  |  |  | 8(1) | 6 |  |  |  |
|  | 17 | 1 | 1 | 5(1) |  |  | 8 | 2(1) |  |  |  |  |  |  |  | 1 | 2 |  |  |  |
|  | 18 | 2 |  |  |  |  | 37(6) | 18(2) |  | 1 |  |  |  |  |  |  |  |  |  |  |
|  | 19 | 1 |  |  |  |  | 10 | 4 |  |  |  |  |  |  |  | 5(1) |  |  |  |  |
|  | 20 | 3(1) |  |  |  |  | 13(4) | 31(7) |  | 1 |  |  |  |  |  | 2(2) |  |  |  |  |
|  | 21 |  |  | 1 |  |  | 17(1) | 10(2) |  | 1 |  |  |  |  |  | 2 | 5 |  |  |  |

Data indicate numbers of lesions. Numbers in parenthesis indicate the number of clinically significant prostate cancers at biopsy.

**V.7. Downgrading and upgrading combinations obtained with PI-RADSv2.1 as compared to PI-RADSv2 in the predefined lesions of the transition zone**

|  | Reader | Downgrading combinations from PI-RADSv2 to PI-RADSv2.1 | | | | | | | | | | | Upgrading combinations from PI-RADSv2 to PI-RADSv2.1 | | | | | | | |
| --- | --- | --- | --- | --- | --- | --- | --- | --- | --- | --- | --- | --- | --- | --- | --- | --- | --- | --- | --- | --- |
|  |  | 2 to 1 | 3 to 1 | 4 to 1 | 5 to 1 | 3 to 1 | 3 to 2 | 4 to 2 | 5 to 2 | 4 to 3 | 5 to 3 | 5 to 4 | 1 to 2 | 1 to 3 | 1 to 4 | 2 to 3 | 2 to 4 | 2 to 5 | 3 to 4 | 4 to 5 |
| Experienced seniors | 1 |  |  |  |  |  | 3 |  |  | 1 |  |  |  |  |  | 2(1) |  |  |  |  |
|  | 2 | 3 |  |  |  |  | 2(1) |  |  | 2(2) |  |  | 1 |  |  |  |  |  |  |  |
|  | 3 | 2(1) |  |  |  |  | 2(1) |  |  |  |  |  |  |  |  |  | 4 |  |  |  |
|  | 4 |  |  |  |  |  | 4 |  |  | 2 |  |  | 1(1) |  |  | 1 |  |  |  |  |
|  | 5 |  |  |  |  |  |  |  |  | 1 |  |  |  |  |  |  |  |  |  |  |
|  | 6 |  |  |  |  |  | 3 |  |  |  |  |  |  |  |  | 4(1) |  |  |  |  |
|  | 7 | 1(1) |  |  |  |  |  |  |  | 4(1) |  |  |  |  |  |  |  |  |  |  |
| Less experienced seniors | 8 | 2(1) |  |  |  |  | 2 |  |  | 4(1) |  |  |  |  |  | 6(2) |  |  |  |  |
|  | 9 |  |  |  |  |  |  |  |  |  |  |  |  |  |  | 2(1) |  |  |  |  |
|  | 10 |  |  |  |  |  | 2 |  |  |  | 1(1) |  |  |  |  |  |  |  |  |  |
|  | 11 | 1 |  |  |  |  |  |  |  |  |  |  |  |  |  |  |  |  |  |  |
|  | 12 | 2 |  |  |  |  | 2 |  |  |  |  |  |  |  |  | 1 |  |  |  |  |
|  | 13 |  |  |  |  |  |  |  |  |  |  |  |  |  |  | 1 |  | 1 |  |  |
|  | 14 |  |  |  |  |  | 1 |  |  | 4(1) |  |  |  |  |  | 2(1) |  |  |  |  |
| Juniors | 15 |  |  |  |  |  |  |  |  |  |  |  |  |  |  | 3 |  |  |  |  |
|  | 16 |  |  |  |  |  | 4 |  |  | 2(1) | 1(1) |  |  |  |  |  |  |  |  |  |
|  | 17 | 1 |  |  |  |  | 1 |  |  |  |  |  |  |  |  | 1 |  |  |  |  |
|  | 18 | 1 |  |  |  |  | 2(6) |  |  | 1 |  |  |  |  |  |  |  |  |  |  |
|  | 19 |  |  |  |  |  |  |  |  |  |  |  |  |  |  | 3 |  |  |  |  |
|  | 20 | 3(1) |  |  |  |  | 1(1) |  |  | 1 |  |  |  |  |  | 2(2) |  |  |  |  |
|  | 21 |  |  |  |  |  | 3(1) |  |  | 1 |  |  |  |  |  |  |  |  |  |  |

Data indicate numbers of lesions. Numbers in parenthesis indicate the number of clinically significant prostate cancers at biopsy.

**VI. Per-lobe analysis**

**VI.1. Additional lesions described by the readers**

|  | Reader | Nb of additional lesions |  |  |  |
| --- | --- | --- | --- | --- | --- |
|  |  | PZ | TZ | CZ | Total |
| Experienced seniors | 1 | 9 | 12 | 4 | 25 |
|  | 2 | 26 | 23 | 6 | 55 |
|  | 3 | 17 | 44 | 3 | 64 |
|  | 4 | 6 | 15 | 0 | 21 |
|  | 5 | 24 | 35 | 7 | 66 |
|  | 6 | 4 | 14 | 7 | 25 |
|  | 7 | 60 | 49 | 18 | 127 |
|  | Median (IQR) | 17 (8-25) | 23 (14-40) | 6 (4-7) | 55 (25-65) |
| Less-experienced seniors | 8 | 6 | 3 | 0 | 9 |
|  | 9 | 27 | 4 | 6 | 37 |
|  | 10 | 54 | 42 | 18 | 114 |
|  | 11 | 11 | 5 | 6 | 22 |
|  | 12 | 29 | 20 | 19 | 68 |
|  | 13 | 17 | 37 | 6 | 60 |
|  | 14 | 31 | 9 | 3 | 43 |
|  | Median (IQR) | 27 (14-30) | 9 (5-29) | 6 (5-12) | 43 (30-64) |
| Juniors | 15 | 27 | 84 | 11 | 122 |
|  | 16 | 12 | 6 | 2 | 20 |
|  | 17 | 60 | 63 | 16 | 139 |
|  | 18 | 20 | 30 | 23 | 73 |
|  | 19 | 38 | 37 | 4 | 79 |
|  | 20 | 22 | 18 | 4 | 44 |
|  | 21 | 16 | 37 | 11 | 64 |
|  | Median (IQR) | 22 (18-33) | 37 (24-50) | 11 (4-14) | 73 (54-101) |
| All readers | Median (IQR) | 22 (12-29) | 23 (12-37) | 6 (4-11) | 60 (25-73) |

Data indicate numbers of lesions.

**VI.2. Detection rates of clinically significant prostate cancer as a function of PI-RADSv2.1 and v2 PI-RADSv2 final scores (per-lobe analysis)**

| Reader | PI-RADSv2.1 | | | | | PI-RADSv2 | | | | |
| --- | --- | --- | --- | --- | --- | --- | --- | --- | --- | --- |
|  | 1 | 2 | 3 | 4 | 5 | 1 | 2 | 3 | 4 | 5 |
| 1 | 7/84 (8%) | 4/61 (7%) | 7/44 (16%) | 40/83 (48%) | 35/46 (76%) | 0/0  (-) | 11/135 (8%) | 6/46 (13%) | 41/91 (45%) | 35/46 (76%) |
| 2 | 6/84 (7%) | 5/51 (10%) | 3/36 (9%) | 36/96 (38%) | 43/51 (84%) | 1/2 (50%) | 6/103 (6%) | 3/34 (9%) | 40/128 (31%) | 43/51 (84%) |
| 3 | 11/99 (11%) | 5/45 (11%) | 5/38 (13%) | 35/79 (44%) | 37/57 (65%) | 1/7 (14%) | 15/128 (12%) | 6/51 (12%) | 34/75 (45%) | 37/57 (65%) |
| 4 | 4/75 (5%) | 4/54 (7%) | 5/27 (19%) | 25/89 (28%) | 55/73 (75%) | 0/2  (0%) | 6/113 (5%) | 5/24 (21%) | 27/106 (25%) | 55/73 (75%) |
| 5 | 0/2  (0%) | 5/65 (8%) | 4/26 (15%) | 29/141 (21%) | 55/84 (65%) | 0/0  (-) | 5/62 (8%) | 4/29 (14%) | 29/143 (20%) | 55/84 (65%) |
| 6 | 0/8  (0%) | 11/135 (8%) | 10/43 (22%) | 37/86 (43%) | 35/46 (76%) | 0/1  (0%) | 10/125 (8%) | 9/47 (19%) | 39/98 (40%) | 35/47 (75%) |
| 7 | 0/6  (0%) | 5/64 (8%) | 2/23 (9%) | 43/156 (28%) | 43/69 (62%) | 0/0  (-) | 3/49 (6%) | 0/17 (0%) | 47/182 (26%) | 43/70 (61%) |
| 8 | 4/56 (7%) | 13/75 (17%) | 6/19 (32%) | 38/125 (30%) | 32/43 (74%) | 0/0  (-) | 9/109 (8%) | 4/15 (27%) | 46/149 (31%) | 34/45 (76%) |
| 9 | 0/0  (-) | 8/95 (8%) | 3/20 (15%) | 40/148 (27%) | 42/55 (76%) | 0/0  (-) | 9/100 (9%) | 2/17 (12%) | 40/146 (27%) | 42/55 (76%) |
| 10 | 1/17 (6%) | 3/52 (6%) | 13/86 (15%) | 37/113 (33%) | 39/50 (78%) | 0/0  (-) | 4/57 (7%) | 12/92 (13%) | 35/116 (30%) | 42/53 (79%) |
| 11 | 12/87 (14%) | 0/12 (0%) | 3/9 (33%) | 33/141 (23%) | 45/69 (65%) | 0/2  (0%) | 11/93 (12%) | 3/9 (33%) | 32/143 (22%) | 47/71 (66%) |
| 12 | 1/50 (2%) | 1/32 (3%) | 0/18 (0%) | 37/136 (27%) | 54/82 (66%) | 0/1  (0%) | 2/63 (3%) | 0/23 (0%) | 37/149 (25%) | 54/82 (66%) |
| 13 | 7/71 (10%) | 1/4 (25%) | 2/18 (11%) | 41/162 (25%) | 42/63 (67%) | 1/6 (17%) | 7/72 (10%) | 2/14 (14%) | 41/164 (25%) | 42/62 (68%) |
| 14 | 0/1  (0%) | 6/105 (6%) | 5/23 (22%) | 37/129 (29%) | 45/60 (75%) | 0/1  (0%) | 7/93 (8%) | 3/25 (12%) | 38/139 (27%) | 45/60 (75%) |
| 15 | 0/0  (-) | 2/42 (5%) | 2/33 (6%) | 39/150 (26%) | 50/93 (54%) | 0/0  (-) | 3/57 (5%) | 1/18 (6%) | 39/150 (26%) | 50/93 (54%) |
| 16 | 4/55 (7%) | 4/41 (10%) | 13/60 (22%) | 36/113 (32%) | 36/49 (73%) | 0/0  (-) | 9/99 (9%) | 10/49 (20%) | 37/118 (31%) | 37/52 (71%) |
| 17 | 5/49 (10%) | 1/15 (7%) | 4/24 (17%) | 35/152 (23%) | 48/78 (62%) | 0/1  (0%) | 4/44 (9%) | 3/28 (11%) | 38/167 (23%) | 48/78 (62%) |
| 18 | 6/68 (9%) | 5/66 (8%) | 11/58 (19%) | 28/67 (42%) | 43/59 (73%) | 0/2  (0%) | 6/82 (7%) | 11/81 (14%) | 33/94 (35%) | 43/59 (73%) |
| 19 | 0/0  (-) | 4/71 (6%) | 3/40 (8%) | 36/117 (31%) | 50/90 (56%) | 0/1  (0%) | 5/68 (7%) | 2/38 (5%) | 36/121 (30%) | 50/90 (56%) |
| 20 | 4/76 (5%) | 16/76 (21%) | 8/42 (19%) | 37/82 (45%) | 28/42 (67%) | 1/4 (25%) | 10/114 (9%) | 8/45 (18%) | 46/113 (41%) | 28/42 (67%) |
| 21 | 7/57 (13%) | 3/41 (7%) | 9/55 (16%) | 39/111 (35%) | 35/54 (65%) | 1/2 (50%) | 7/72 (10%) | 10/72 (14%) | 40/118 (34%) | 35/54 (65%) |

**VI.3.** **Areas under the receiver operating characteristic curves obtained by the readers using PI-RADSv2 and PI-RADSv2.1 (per-lobe analysis)**

|  | **Reader** | **PI-RADS v2.1** |  | **PI-RADS v2** |  |
| --- | --- | --- | --- | --- | --- |
|  |  | **AUC [95% CI]** | p^(1)^ | **AUC [95% CI]** | p^(1)^ |
| Experienced seniors | 1 | 0.83 [0.79-0.87] |  | 0.84 [0.84-0.88] |  |
|  | 2 | 0.85 [0.81-0.89] |  | 0.83 [0.80-0.89] |  |
|  | 3 | 0.78 [0.73-0.83] |  | 0.77 [0.72-0.83] |  |
|  | 4 | 0.86 [0.82-0.89] |  | 0.86 [0.82-0.89] |  |
|  | 5 | 0.81 [0.76-0.86] |  | 0.80 [0.76-0.85] |  |
|  | 6 | 0.84 [0.79-0.87] |  | 0.82 [0.78-0.86] |  |
|  | 7 | 0.78 [0.73-0.83] |  | 0.78 [0.73-0.83] |  |
|  | Mean | 0.82 [0.79-0.86] | **0.03** | 0.82 [0.79-0.86] | **0.003** |
| Less-experienced seniors | 8 | 0.77 [0.72-0.81] |  | 0.80 [0.75-0.84] |  |
|  | 9 | 0.81 [0.77-0.86] |  | 0.81 [0.76-0.85] |  |
|  | 10 | 0.82 [0.77-0.86] |  | 0.82 [0.78-0.86] |  |
|  | 11 | 0.76 [0.70-0.80] |  | 0.77 [0.71-0.82] |  |
|  | 12 | 0.85 [0.79-0.88] |  | 0.84 [0.79-0.87] |  |
|  | 13 | 0.77 [0.72-0.82] |  | 0.77 [0.72-0.82] |  |
|  | 14 | 0.83 [0.79-0.88] |  | 0.82 [0.78-0.86] |  |
|  | Mean | 0.79 [0.76-0.83] | 0.71 | 0.80 [0.76-0.83] | 0.23 |
| Juniors | 15 | 0.77 [0.71-0.82] |  | 0.77 [0.71-0.82] |  |
|  | 16 | 0.79 [0.74-0.83] |  | 0.78 [0.74-0.83] |  |
|  | 17 | 0.77 [0.71-0.82] |  | 0.77 [0.72-0.82] |  |
|  | 18 | 0.82 [0.78-0.86] |  | 0.82 [0.78-0.86] |  |
|  | 19 | 0.78 [0.73-0.83] |  | 0.78 [0.73-0.82] |  |
|  | 20 | 0.78 [0.74-0.82] |  | 0.76 [0.70-0.82] |  |
|  | 21 | 0.76 [0.71-0.80] |  | 0.74 [0.70-0.80] |  |
|  | Mean | 0.79 [0.75-0.83] | - | 0.78 [0.74-0.82] | - |

(1) p value comparing the AUC of experienced and less-experienced seniors to that of juniors. Bold characters indicate statistical significance.

PI-RADSv2: prostate imaging reporting and data system version 2; PI-RADSv2.1: prostate imaging reporting and data system version 2.1; AUC: area under the receiver operating characteristic curve; 95% CI: 95% confidence interval.

**VI.4. Sensitivities and specificities obtained by the readers using PI-RADSv2.1 (per-lobe analysis)**

| * | Reader | Threshold ≥3 | | Threshold ≥4 | |
| --- | --- | --- | --- | --- | --- |
|  |  | Se [95% CI] | Sp [95% CI] | Se [95% CI] | Sp [95% CI] |
| Experienced seniors | 1 | 88 [80-94] | 60 [53-66] | 81 [71-88] | 76 [70-81] |
|  | 2 | 88 [80-94] | 55 [48-62] | 85 [76-92] | 70 [63-75] |
|  | 3 | 83 [74-90] | 57 [50-63] | 77 [68-85] | 72 [65-77] |
|  | 4 | 91 [84-96] | 54 [47-60] | 86 [77-92] | 64 [57-70] |
|  | 5 | 95 [88-98] | 28 [21.8-34] | 90 [82-95] | 37 [31-44] |
|  | 6 | 88 [80-94] | 60 [52.8-66] | 77 [68-85] | 73 [67-79] |
|  | 7 | 95 [88-98] | 55 [48-62] | 92 [85-97] | 38 [32-45] |
|  | Pooled | 90 [86-94] | 48 [39-58] | 85 [78-90] | 62 [51-72] |
| Less-experienced seniors | 8 | 82 [72-89] | 51 [44-57] | 75 [65-84] | 56 [50-63] |
|  | 9 | 91 [84-96] | 39 [38-45] | 88 [80-94] | 46 [40-53] |
|  | 10 | 96 [89-99] | 29 [23-35] | 82 [72-89] | 61 [55-68] |
|  | 11 | 87 [79-93] | 39 [32-45] | 84 [75-91] | 41 [35-48] |
|  | 12 | 98 [92-100] | 36 [29-42] | 98 [92-100] | 44 [37-50] |
|  | 13 | 91 [84-96] | 30 [24-36] | 89 [81-95] | 37 [31-44] |
|  | 14 | 94 [86-98] | 44 [38-51] | 88 [80-94] | 52 [46-59] |
|  | Pooled | 92 [88-95] | 38 [29-47] | 87 [81-92] | 48 [37-59] |
| Juniors | 15 | 98 [92-100] | 18 [13-23] | 96 [89-99] | 32 [26-38] |
|  | 16 | 91 [84-96] | 39 [33-46] | 77 [68-85] | 60 [53-66] |
|  | 17 | 94 [86-98] | 26 [20-32] | 89 [82-95] | 35 [28-41] |
|  | 18 | 88 [80-94] | 55 [48-61] | 76 [66-85] | 76 [69-81] |
|  | 19 | 96 [89-99] | 30 [24-36] | 92 [85-97] | 46 [40-53] |
|  | 20 | 78 [67-86] | 59 [52-65] | 70 [60-79] | 74 [68-79] |
|  | 21 | 89 [81-95] | 39 [33-46] | 79 [70-87] | 60 [53-66] |
|  | Pooled | 92 [87.3-94.7] | 37 [28-46] | 84 [78-90] | 55 [44-66] |

Sensitivities and specificities are expressed in percentages.

Se: sensitivity; Spe: specificity. 95% CI: 95% confidence interval.

**VI.5. Sensitivities and specificities obtained by the readers using PI-RADSv2 (per-lobe analysis)**

|  | Reader | Threshold ≥3 | | Threshold ≥4 | |
| --- | --- | --- | --- | --- | --- |
|  |  | Se [95% CI] | Spe [95% CI] | Se [95% CI] | Spe [95% CI] |
| Experienced seniors | 1 | 88 [80-94] | 55 [48-62] | 82 [72-89] | 73 [67-79] |
|  | 2 | 92 [85-97] | 44 [37-50] | 89 [81-95] | 57 [51-64] |
|  | 3 | 83 [74-90] | 53 [46-60] | 76 [66-85] | 73 [67-79] |
|  | 4 | 94 [86-98] | 48 [42-55] | 88 [80-94] | 57 [50-63] |
|  | 5 | 95 [88-98] | 25 [20-32] | 90 [82-95] | 36 [30-43] |
|  | 6 | 89 [81-95] | 52 [45-58] | 80 [70-87] | 68 [62-74] |
|  | 7 | 97 [91-99] | 20 [15-26] | 97 [91-99] | 28 [22-34] |
|  | Mean | 91 [88-94] | 42 [34-50] | 87 [81-91] | 56 [46-67] |
| Less experienced seniors | 8 | 90 [82-95] | 44 [38-51] | 86 [77-92] | 49 [43-56] |
|  | 9 | 90 [82-95] | 40 [34-47] | 88 [80-94] | 47 [40-54] |
|  | 10 | 96 [89-99] | 24 [18-30] | 83 [74-90] | 59 [52-66] |
|  | 11 | 88 [80-94] | 37 [31-44] | 85 [76-92] | 40 [34-47] |
|  | 12 | 98 [92-100] | 28 [22-34] | 98 [92-100] | 38 [31-44] |
|  | 13 | 91 [84-96] | 31 [25-38] | 89 [81-95] | 36 [30-43] |
|  | 14 | 92 [85-97] | 39 [32-45] | 89 [81-95] | 48 [42-55] |
|  | Mean | 93 [90-95] | 34 [27-43] | 89 [84-93] | 45 [35-56] |
| Juniors | 15 | 97 [91-99] | 24 [19-30] | 96 [89-99] | 32 [26-38] |
|  | 16 | 90 [83-95] | 40 [34-47] | 80 [70-87] | 57 [51-64] |
|  | 17 | 96 [89-99] | 18.2 [13-24] | 92 [85-97] | 29 [23-36] |
|  | 18 | 94 [86-98] | 35 [28-41] | 82 [72-89] | 66 [59-72] |
|  | 19 | 95 [88-98] | 28 [23-35] | 95 [85-97] | 44 [38-51] |
|  | 20 | 88 [80-94] | 48 [41-54] | 80 [70-87] | 64 [57-70] |
|  | 21 | 91 [84-96] | 29 [23-36] | 81 [71-88] | 57 [50-63] |
|  | Mean | 93 [90-95] | 31 [24-39] | 87 [81-91] | 50 [39-60] |

Sensitivities and specificities are expressed in percentages.

Se: sensitivity; Spe: specificity. 95% CI: 95% confidence interval.

**VI.6. Downgrading and upgrading combinations obtained with PI-RADSv2.1 as compared to PI-RADSv2 (per-lobe analysis)**

|  | Reader | Downgrading combinations from PI-RADSv2 to PI-RADSv2.1 | | | | | | | | | | Upgrading combinations from PI-RADSv2 to PI-RADSv2.1 | | | | | |
| --- | --- | --- | --- | --- | --- | --- | --- | --- | --- | --- | --- | --- | --- | --- | --- | --- | --- |
|  |  | 2 to 1 | 3 to 1 | 4 to 1 | 5 to 1 | 3 to 2 | 4 to 2 | 5 to 2 | 4 to 3 | 5 to 3 | 5 to 4 | 1 to 2 | 1 to 3 | 1 to 4 | 2 to 3 | 2 to 4 | 3 to 4 |
| Experienced seniors | 1 | 83 (7) | 1 (0) |  |  | 5 (0) | 7 (2) |  | 2 (0) |  |  |  |  |  | 2 (0) | 1 (1) |  |
|  | 2 | 78 (5) | 1 (0) | 4 (0) |  | 5 (2) | 27 (2) |  | 7 (2) |  |  | 1 (0) |  |  | 3 (0) | 4 (0) | 2 (0) |
|  | 3 | 92 (10) |  |  |  | 15 (2) |  |  |  |  |  |  |  |  | 3 (1) | 3 (1) | 1 (0) |
|  | 4 | 73 (4) |  |  |  | 4 (0) | 14 (2) |  | 3 (0) |  |  |  |  |  | 4 (0) |  |  |
|  | 5 | 2 (0) |  |  |  | 1 (0) | 4 (0) |  | 2 (0) |  |  |  |  |  |  |  | 4 (0) |
|  | 6 | 3 (0) | 4 (0) |  |  | 14 (1) | 11 (2) |  | 2 (0) | 1 (0) |  |  |  |  | 11 (2) | 1 (0) |  |
|  | 7 | 6 (0) |  |  |  | 4 (0) | 18 (2) | 1 (0) | 8 (2) |  |  |  |  |  | 2 (0) |  |  |
| Less experienced seniors | 8 | 56 (4) |  |  |  | 5 (1) | 23 (7) | 2 (2) | 4 (1) |  |  |  |  |  | 5 (2) | 3 (0) |  |
|  | 9 |  |  |  |  |  |  |  |  |  |  |  |  |  | 3 (1) | 2 (0) |  |
|  | 10 | 16 (1) |  | 1 (0) |  | 11 (0) | 2 (0) |  | 3 (0) |  | 2 (2) |  |  |  | 1 (0) | 1 (0) |  |
|  | 11 | 79 (9) |  | 4 (1) | 2 (2) |  |  |  |  |  |  |  |  |  |  | 2 (2) |  |
|  | 12 | 40 (1) | 4 (0) | 5 (0) |  | 6 (0) | 6 (0) |  | 2 (0) |  |  |  |  |  | 3 (0) |  |  |
|  | 13 | 65 (6) |  |  |  |  | 1 (0) |  |  |  |  |  |  | 1 (0) | 4 (0) |  |  |
|  | 14 |  |  |  |  | 11 (0) | 6 (0) |  | 6 (1) |  |  |  |  |  | 3 (1) | 2 (0) |  |
| Juniors | 15 |  |  |  |  |  |  |  |  |  |  |  |  |  | 15 (1) |  |  |
|  | 16 | 55 (4) |  |  |  | 4 (0) | 5 (1) |  | 8 (1) | 1 (1) | 2 (0) |  |  |  | 6 (1) | 6 (1) |  |
|  | 17 | 37 (3) | 3 (0) | 8 (2) |  | 7 (0) | 3 (0) |  | 5 (1) |  |  |  |  |  | 1 (0) | 1 (0) |  |
|  | 18 | 66 (6) |  |  |  | 34 (2) | 18 (3) |  | 9 (2) |  |  |  |  |  | 2 (0) |  |  |
|  | 19 |  |  |  |  |  | 8 (0) |  | 1 (0) |  |  |  |  |  | 6 (1) |  | 1 (0) |
|  | 20 | 72 (3) |  |  |  | 9 (2) | 26 (8) |  | 5 (1) |  |  |  |  |  | 1 (1) |  |  |
|  | 21 | 53 (6) |  | 2 (0) |  | 20 (1) | 7 (1) |  | 3 (0) |  |  |  |  |  | 2 (0) | 3 (0) | 2 (0) |

Data indicate numbers of lesions. Numbers in parenthesis indicate the number of clinically significant prostate cancers at biopsy.

**VII. Per-patient analysis**

**VII.1. Detection rates of clinically significant prostate cancer as a function of PI-RADSv2.1 and v2 PI-RADSv2 final scores (per-patient analysis)**

| Reader | PI-RADSv2.1 | | | | | PI-RADSv2 | | | | |
| --- | --- | --- | --- | --- | --- | --- | --- | --- | --- | --- |
|  | 1 | 2 | 3 | 4 | 5 | 1 | 2 | 3 | 4 | 5 |
| 1 | 0/5  (0%) | 2/31 (6%) | 4/27 (15%) | 29/61 (48%) | 28/35 (80%) | 0/0  (-) | 1/31 (3%) | 4/27 (15%) | 30/66 (45%) | 28/35 (80%) |
| 2 | 0/12 (0%) | 2/23 (9%) | 2/19 (11%) | 23/64 (36%) | 36/41 (88%) | 0/0  (-) | 1/22 (5%) | 1/15 (7%) | 25/81 (31%) | 36/41 (88%) |
| 3 | 2/26 (8%) | 2/18 (11%) | 4/25 (16%) | 26/51 (51%) | 29/39 (74%) | 0/2  (0%) | 5/39 (13%) | 4/32 (13%) | 25/47 (53%) | 29/39 (74%) |
| 4 | 0/11 (0%) | 1/20 (5%) | 3/14 (21%) | 17/62 (27%) | 42/52 (81%) | 0/0  (-) | 0/24 (0%) | 3/10 (30%) | 18/73 (25%) | 42/52 (81%) |
| 5 | 0/0  (-) | 0/13 (0%) | 1/9 (11%) | 20/82 (24%) | 42/55 (76%) | 0/0  (-) | 0/11 (0%) | 1/8 (13%) | 20/85 (24%) | 42/55 (76%) |
| 6 | 0/0  (-) | 1/36 (3%) | 5/30 (17%) | 31/62 (50%) | 26/31 (84%) | 0/0  (-) | 1/34 (3%) | 5/26 (19%) | 31/67 (46%) | 26/32 (81%) |
| 7 | 0/0  (-) | 1/9 (11%) | 0/10 (0%) | 29/88 (33%) | 33/52 (63%) | 0/0  (-) | 0/5  (0%) | 0/6  (0%) | 30/96 (31%) | 33/52 (63%) |
| 8 | 0/5  (0%) | 3/24 (13%) | 2/7 (29%) | 32/89 (36%) | 26/34 (76%) | 0/0  (-) | 0/19 (0%) | 1/5 (20%) | 35/100 (35%) | 27/35 (77%) |
| 9 | 0/0  (-) | 0/14 (0%) | 1/11 (9%) | 26/88 (30%) | 36/46 (78%) | 0/0  (-) | 0/17 (0%) | 1/9 (11%) | 26/87 (30%) | 36/46 (78%) |
| 10 | 0/0  (-) | 0/6  (0%) | 4/38 (11%) | 27/76 (36%) | 32/39 (82%) | 0/0  (-) | 0/4  (0%) | 4/39 (10%) | 25/75 (33%) | 34/41 (83%) |
| 11 | 1/12 (8%) | 0/3  (0%) | 2/3 (66%) | 25/89 (28%) | 35/52 (67%) | 0/0  (-) | 1/14 (7%) | 2/3 (66%) | 24/89 (27%) | 36/53 (68%) |
| 12 | 0/6  (0%) | 0/7  (0%) | 0/10 (0%) | 20/73 (27%) | 43/63 (68%) | 0/0  (-) | 0/11 (0%) | 0/8  (0%) | 20/77 (26%) | 43/63 (68%) |
| 13 | 0/2  (0%) | 0/2  (0%) | 1/8 (13%) | 26/95 (27%) | 36/52 (69%) | 0/0  (-) | 0/6  (0%) | 1/5 (20%) | 26/96 (27%) | 36/52 (69%) |
| 14 | 0/0  (-) | 0/21 (0%) | 2/11 (18%) | 26/83 (31%) | 35/44 (80%) | 0/0  (-) | 0/12 (0%) | 0/13 (0%) | 28/90 (31%) | 35/44 (80%) |
| 15 | 0/0  (-) | 0/1  (0%) | 0/14 (0%) | 23/77 (30%) | 40/67 (60%) | 0/0  (-) | 0/7  (0%) | 0/8  (0%) | 23/77 (30%) | 40/67 (60%) |
| 16 | 0/4  (0%) | 0/10 (0%) | 7/29 (24%) | 27/80 (34%) | 29/36 (81%) | 0/0  (-) | 0/13 (0%) | 6/26 (23%) | 27/82 (33%) | 30/38 (79%) |
| 17 | 1/4 (25%) | 0/3  (0%) | 1/8 (13%) | 24/88 (27%) | 37/56 (66%) | 0/0  (-) | 0/3  (0%) | 1/9 (11%) | 25/91 (27%) | 37/56 (66%) |
| 18 | 0/5  (0%) | 2/30 (7%) | 2/30 (7%) | 23/48 (48%) | 36/46 (78%) | 0/0  (-) | 0/15 (0%) | 2/33 (6%) | 25/65 (38%) | 36/46 (78%) |
| 19 | 0/0  (-) | 0/7  (0%) | 0/20 (0%) | 21/63 (33%) | 42/69 (61%) | 0/0  (-) | 0/4  (0%) | 0/20 (0%) | 21/66 (32%) | 42/69 (61%) |
| 20 | 0/10 (0%) | 5/33 (17%) | 5/25 (20%) | 31/59 (53%) | 22/32 (69%) | 0/0  (-) | 0/23 (0%) | 4/26 (15%) | 37/78 (47%) | 22/32 (68%) |
| 21 | 0/0  (-) | 1/15 (7%) | 4/26 (15%) | 31/77 (40%) | 27/41 (66%) | 0/0  (-) | 0/7  (0%) | 4/33 (12%) | 32/78 (41%) | 27/41 (66%) |

**VII.2. Areas under the receiver operating characteristic curves obtained by the readers using PI-RADSv2 and PI-RADSv2.1 (per-patient analysis)**

|  | **Reader** | **PI-RADS v2.1** | **PI-RADS v2** |
| --- | --- | --- | --- |
| Experienced seniors | 1 | 0.84 [0.79-0.89] | 0.85 [0.79-0.89] |
|  | 2 | 0.88 [0.84-0.92] | 0.88 [0.83-0.91] |
|  | 3 | 0.81 [0.76-0.87] | 0.81 [0.75-0.86] |
|  | 4 | 0.87 [0.82-0.91] | 0.86 [0.81-0.91] |
|  | 5 | 0.86 [0.77-0.88] | 0.85 [0.76-0.88] |
|  | 6 | 0.86 [0.82-0.91] | 0.85 [0.79-0.90] |
|  | 7 | 0.74 [0.67-0.79] | 0.69 [0.64-0.74] |
|  | Mean | 0.85 [0.81-0.89] | 0.87 [0.81-0.98] |
| Less experienced seniors | 8 | 0.78 [0.72-0.84] | 0.82 [0.71-0.85] |
|  | 9 | 0.85 [0.75-0.88] | 0.85 [0.75-0.88] |
|  | 10 | 0.84 [0.78-0.89] | 0.84 [0.79-0.90] |
|  | 11 | 0.76 [0.69-0.82] | 0.76 [0.70-0.82] |
|  | 12 | 0.77 [0.73-0.82] | 0.77 [0.72-0.82] |
|  | 13 | 0.78 [0.69-0.82] | 0.78 [0.69-0.81] |
|  | 14 | 0.84 [0.78-0.89] | 0.79 [0.74-0.83] |
|  | Mean | 0.80 [0.76-0.85] | 0.84 [0.77-0.99] |
| Juniors | 15 | 0.71 [0.65-0.76] | 0.70 [0.65-0.76] |
|  | 16 | 0.78 [0.72-0.85] | 0.79 [0.72-0.85] |
|  | 17 | 0.76 [0.69-0.81] | 0.77 [0.68-0.81] |
|  | 18 | 0.86 [0.82-0.91] | 0.86 [0.81-0.90] |
|  | 19 | 0.74 [0.68-0.79] | 0.73 [0.68-0.78] |
|  | 20 | 0.77 [0.71-0.83] | 0.79 [0.73-0.85] |
|  | 21 | 0.75 [0.69-0.81] | 0.76 [0.70-0.82] |
|  | Mean | 0.80 [0.75-0.84] | 0.83 [0.76-0.99] |

**VII.3. Sensitivities and specificities obtained by the readers using PI-RADSv2.1 (per-patient analysis)**

|  | Reader # | Threshold ≥3 | | Threshold ≥4 | |
| --- | --- | --- | --- | --- | --- |
|  |  | Se [95% CI] | Spe [95% CI] | Se [95% CI] | Spe [95% CI] |
| Experienced seniors | 1 | 97 [89-100] | 35 [26-46] | 90 [80-96] | 59 [49-69] |
|  | 2 | 97 [89-100] | 34 [25-45] | 94 [85-98] | 52 [42-62] |
|  | 3 | 94 [85-98] | 42 [32-52] | 87 [77-94] | 64 [53-73] |
|  | 4 | 98 [91-100] | 31 [22-42] | 94 [85-98] | 43 [33-53] |
|  | 5 | 100 [94-100] | 14 [7-22] | 98 [91-100] | 22 [14-31] |
|  | 6 | 98 [91-100] | 36 [27-47] | 90 [80-96] | 63 [52-72] |
|  | 7 | 98 [91-100] | 8 [4-16] | 98 [91-100] | 19 [12-28] |
|  | Mean | 98 [95-99] | 27 [16-41] | 94 [89-97] | 45 [31-59] |
| Less experienced seniors | 8 | 95 [87-99] | 27 [19-37] | 92 [82-97] | 32 [23-43] |
|  | 9 | 100 [94-100] | 15 [8-23] | 98 [91-100] | 25 [17-35] |
|  | 10 | 100 [94-100] | 6 [2-13] | 94 [85-98] | 42 [32-52] |
|  | 11 | 98 [91-100] | 15 [8-23] | 95 [87-99] | 16 [9-24] |
|  | 12 | 100 [94-100] | 14 [7-22] | 100 [94-100] | 24 [16-34] |
|  | 13 | 100 [94-100] | 4 [1-10] | 98 [91-100] | 11 [6-20] |
|  | 14 | 100 [94-100] | 22 [14-31] | 97 [89-100] | 31 [22-42] |
|  | Mean | 99 [98-100] | 13 [7-22] | 97 [94-99] | 25 [15-37] |
| Juniors | 15 | 100 [94-100] | 1 [0-6] | 100 [94-100] | 16 [9-24] |
|  | 16 | 100 [94-100] | 15 [8-23] | 89 [78-95] | 378 [28-48] |
|  | 17 | 98 [91-100] | 6 [2-13] | 97 [89-100] | 14 [7-22] |
|  | 18 | 97 [89-100] | 34 [25-45] | 94 [85-98] | 64 [53-73] |
|  | 19 | 100 [94-100] | 7 [3-15] | 100 [94-100] | 28 [19-38] |
|  | 20 | 92 [82-97] | 40 [30-50] | 84 [73-92] | 60 [50-70] |
|  | 21 | 98 [91-100] | 15 [8-23] | 92 [82-97] | 38 [28-48] |
|  | Mean | 98 [96-100] | 13 [7-22] | 95 [91-97] | 35 [23-48] |

Sensitivities and specificities are expressed in percentages.

Se: sensitivity; Spe: specificity. 95% CI: 95% confidence interval.

**VII.4. Sensitivities and specificities obtained by the readers using PI-RADSv2 (per-patient analysis)**

|  | Reader # | Threshold ≥3 | | Threshold ≥4 | |
| --- | --- | --- | --- | --- | --- |
|  |  | Se [95% CI] | Spe [95% CI] | Se [95% CI] | Spe [95% CI] |
| Experienced seniors | 1 | 98 [91-100] | 31 [22-41] | 92 [82-97] | 55 [45-65] |
|  | 2 | 98 [91-100] | 22 [14-31] | 97 [89-100] | 36 [27-47] |
|  | 3 | 92 [82-97] | 38 [28-48] | 86 [75-93] | 67 [56-76] |
|  | 4 | 100 [94-100] | 25 [17-35] | 95 [87-99] | 33 [23-43] |
|  | 5 | 100 [94-100] | 11 [6-20] | 98 [91-100] | 19 [12-28] |
|  | 6 | 98 [91-100] | 34 [25-45] | 90 [80-96] | 56 [46-66] |
|  | 7 | 100 [94-100] | 5 [2-12] | 100 [94-100] | 11 [6-20] |
|  | Mean | 99 [ND-ND]^(1)^ | 22 [14-32] | 95 [91-98] | 38 [26-51] |
| Less experienced seniors | 8 | 100 [94-100] | 20 [12-29] | 98 [91-100] | 24 [16-34] |
|  | 9 | 100 [94-100] | 18 [11-27] | 98 [91-100] | 26 [18-36] |
|  | 10 | 100 [94-100] | 4 [1-10] | 94 [85-98] | 41 [31-51] |
|  | 11 | 98 [91-100] | 14 [7-22] | 95 [87-99] | 15 [8-23] |
|  | 12 | 100 [94-100] | 11 [6-20] | 100 [94-100] | 20 [12-29] |
|  | 13 | 100 [94-100] | 6 [2-13] | 98 [91-100] | 10 [5-18] |
|  | 14 | 100 [94-100] | 13 [7-21] | 100 [94-100] | 26 [18-36] |
|  | Mean | 99.9 [ND-ND]^(1)^ | 11 [7-18] | 98 [96-99] | 22 [14-33] |
| Juniors | 15 | 100 [94-100] | 7 [3-14] | 100 [94-100] | 16 [9-24] |
|  | 16 | 100 [94-100] | 14 [7-22] | 90 [80-96] | 34 [25-45] |
|  | 17 | 100 [94-100] | 3 [1-9] | 98 [91-100] | 11 [6-20] |
|  | 18 | 100 [94-100] | 16 [9-24] | 97 [89-100] | 48 [38-58] |
|  | 19 | 100 [94-100] | 4 [1-10] | 100 [94-100] | 25 [17-35] |
|  | 20 | 100 [94-100] | 24 [16-34] | 94 [85-98] | 47 [37-57] |
|  | 21 | 100 [94-100] | 7 [3-14] | 94 [85-98] | 38 [28-48] |
|  | Mean | 100 [ND-ND]^(1)^ | 9 [6-15] | 97 [94-99] | 29 [19-42] |

(1) 95% confidence intervals could not be computed due to the lack of convergence of the model.

Sensitivities and specificities are expressed in percentages.

Se: sensitivity; Spe: specificity. 95% CI: 95% confidence interval.

**VII.5. Inter-reader agreement in the three groups of readers (per-patient analysis)**

|  |  | Overall score |
| --- | --- | --- |
|  |  |  |
| PI-RADSv2.1 | All readers | 0.51 [0.47-0.55] |
|  | Experienced seniors | 0.55 [0.50-0.59] |
|  | Less experienced seniors | 0.56 [0.50-0.61] |
|  | Juniors | 0.46 [0.41-0.50] |
| PI-RADSv2 | All readers | 0.53 [0.49-0.57] |
|  | Experienced seniors | 0.54 [0.49-0.59] |
|  | Less experienced seniors | 0.60 [0.55-0.65] |
|  | Juniors | 0.49 [0.44-0.53] |
